# DOT1L cooperates with the c-Myc-p300 complex to epigenetically derepress *CDH1* transcription factors in breast cancer progression

**DOI:** 10.1038/ncomms8821

**Published:** 2015-07-22

**Authors:** Min-Hyung Cho, Ji-Hye Park, Hee-Joo Choi, Mi-Kyung Park, Hee-Young Won, Yeon-Ji Park, Chang Hoon Lee, Seung-Hyun Oh, Young-Soo Song, Hyun Sung Kim, Young-Ha Oh, Jeong-Yeon Lee, Gu Kong

**Affiliations:** 1 Department of Pathology, College of Medicine, Hanyang University, Seoul 133-791, Korea; 2 Institute for Bioengineering and Biopharmaceutical Research (IBBR), Hanyang University, Seoul 133-791, Korea; 3 College of Pharmacy, Dongguk University, Seoul 100-715, Korea; 4 College of Pharmacy, Gachon University, Incheon 405-840, Republic of Korea

## Abstract

DOT1L has emerged as an anticancer target for MLL-associated leukaemias; however, its functional role in solid tumours is largely unknown. Here we identify that DOT1L cooperates with c-Myc and p300 acetyltransferase to epigenetically activate epithelial–mesenchymal transition (EMT) regulators in breast cancer progression. DOT1L recognizes *SNAIL*, *ZEB1* and *ZEB2* promoters via interacting with the c-Myc-p300 complex and facilitates lysine-79 methylation and acetylation towards histone H3, leading to the dissociation of HDAC1 and DNMT1 in the regions. The upregulation of these EMT regulators by the DOT1L-c-Myc-p300 complex enhances EMT-induced breast cancer stem cell (CSC)-like properties. Furthermore, *in vivo* orthotopic xenograft models show that DOT1L is required for malignant transformation of breast epithelial cells and breast tumour initiation and metastasis. Clinically, DOT1L expression is associated with poorer survival and aggressiveness of breast cancers. Collectively, we suggest that cooperative effect of DOT1L and c-Myc-p300 is critical for acquisition of aggressive phenotype of breast cancer by promoting EMT/CSC.

## Abstract (web summary)

DOT1L is an anti-cancer therapeutic target in leukaemia but has a poorly understood role in solid tumours. Here the authors show that DOT1L expression is associated with poor survival and aggressive cancers by helping to epigenetically activate the epithelial-mesenchymal transition during breast cancer progression.

## Graphical abstract

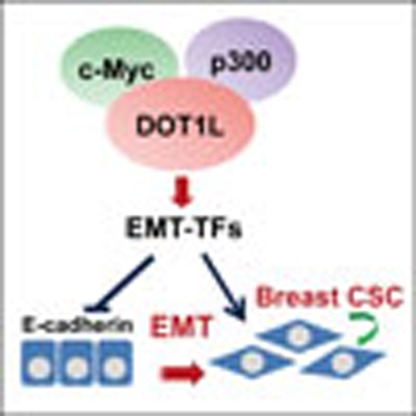

Disruptor of telomeric silencing-1-like (DOT1L) is a class I-like S-adenosyl-L-methionine (SAM)-binding methyltransferase that catalyses the methylation of histone H3 on lysine-79 (H3K79me), an active transcription mark^1^. It plays a crucial role in gene regulatory processes including telomeric silencing and transcription elongation by interplaying with other genetic and epigenetic components such as RNA polymerase (pol) II and E3 ubiquitin ligase of histone H2B (refs 1, 2, 3). Recent studies also show a novel role of DOT1L in regulation of DNA repair and cell cycle progression^4^,^5^. Furthermore, several studies report that DOT1L is crucial for tumour development, especially leukaemogenesis, by associating with mixed lineage leukemia (MLL) fusion proteins and participating in the activation of a leukaemic transcriptional programme^6^,^7^,^8^. Thus, DOT1L has been considered as a potentially important therapeutic target for leukaemia^9^. A very recent study also shows that pharmacologic inhibition of H3K79 methylation suppresses self-renewal of breast cancer stem cell (CSC), breast cancer proliferation, migration and invasion^10^, suggesting DOT1L to be a potential therapeutic target for breast cancer. However, functional mechanism of the oncogenic potential and clinical relevance of DOT1L in solid tumours including breast cancer remain still unclear.

Growing evidence has emerged that the epithelial–mesenchymal transition (EMT), an essential developmental process characterized by loss of cell adhesion, downregulation of E-cadherin and increased cell motility, is closely linked to the generation of stem-like cells^11^,^12^. EMT has similar stem cell characteristics that regulate tumour invasion, metastasis and therapeutic resistance in cancer cells^13^,^14^. Indeed, main CSC regulators including c-Myc, Bmi-1 and Wnt signalling are commonly linked to regulation of EMT^15^,^16^,^17^,^18^,^19^. Furthermore, several EMT-transcription factors (EMT-TFs), such as Twist1, Snail (SNAI1), Slug (SNAI2), ZEB1 and ZEB2 (SIP1), which repress the transcription of *CDH1* encoding E-cadherin, have been shown to play a crucial role in the acquisition of stem cell characteristics^11^,^20^,^21^, implying an important association between EMT and CSCs. Recent studies also show that epigenetic regulators cooperate with EMT-TFs to repress E-cadherin transcription. For instance, Snail and Twist affect epigenetic alteration of E-cadherin expression by collaborating with distinct epigenetic modifiers such as histone methyltransferase, G9a and SET8 as well as histone deacetylases (HDACs) and DNA methyltransferases (DNMTs)^22^,^23^. Conversely, the epigenetic regulatory mechanism of EMT-TF expression remains largely unknown, while epigenetic and transcriptional regulation of E-cadherin has been well described. A recent genome-wide study shows that several EMT-TFs are targeted by DOT1L-associated H3K79me during the reprogramming of embryonic stem cells^24^. More recently, Zhang *et al.*^10^ also demonstrates that pharmacological inhibition of DOT1L enzymatic activity affects E-cadherin, Snail and ZEB1 mRNA expression. However, the functional role with associated mechanism of DOT1L in the regulation of EMT and CSCs still remains to be elucidated.

In this study, we find that DOT1L forms a novel transcriptional active complex with c-Myc and p300 to enhance epigenetic derepression of EMT-TFs and consequently promote EMT-induced CSC properties in human breast cancer. Furthermore, we provide the clinical and *in vivo* evidence that DOT1L is associated with aggressive phenotypes of breast cancer. Therefore, our findings demonstrate functional role of potential oncogene DOT1L in promoting multistep breast carcinogenesis associated with EMT and stem cell-like phenotype as a novel epigenetic regulator of EMT-TFs, suggesting DOT1L to be a promising target for aggressive breast cancer therapy.

## Results

### DOT1L promotes EMT-induced breast tumour metastasis

**Figure 1:**
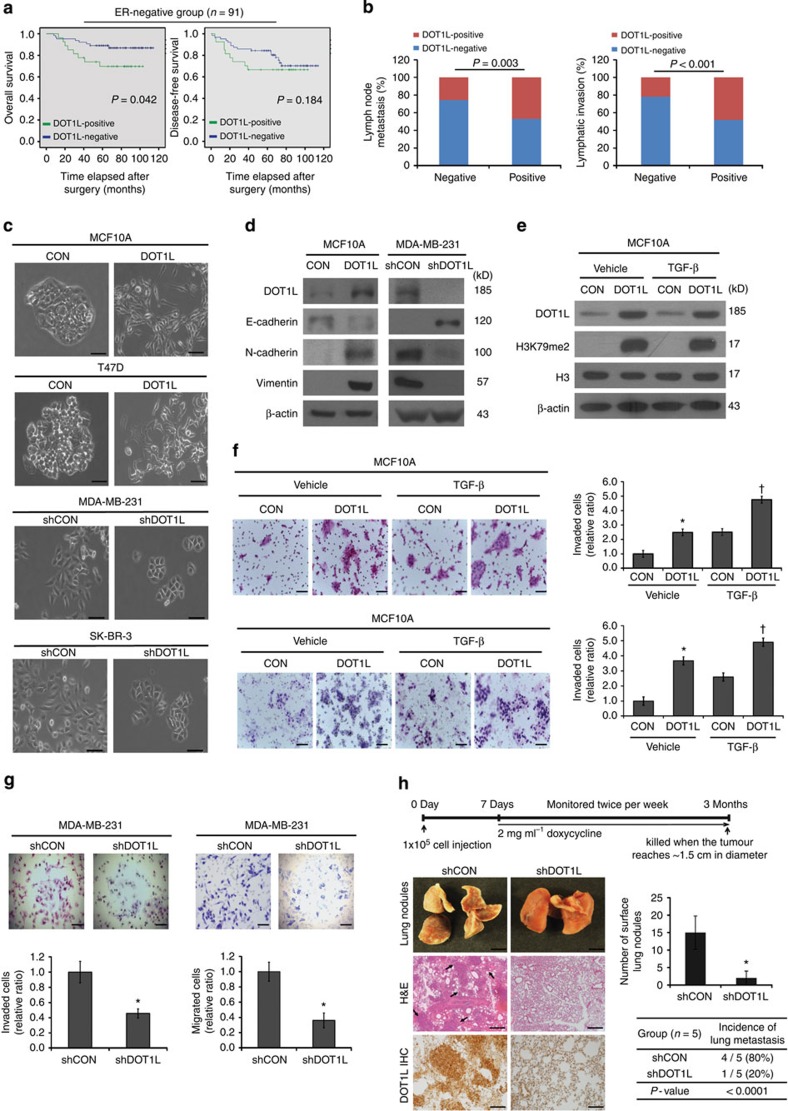
DOT1L is associated with aggressive breast cancer by promoting EMT-induced breast tumour metastasis. (**a**) Analysis of overall and disease-free survival according to DOT1L expression in 91 ER-negative human breast cancers using Kaplan–Meier method with log-rank test. (**b**) The quantification of the association with DOT1L expression and invasion and metastasis in 182 human breast cancers. The *P* values were calculated by *χ*^2^-test. (**c**) Bright-field images of the indicated cell types for observation of changes in cell morphology after DOT1L overexpression and knockdown using lentiviruses encoding DOT1L cDNA or Tet-inducible DOT1L shRNA (shDOT1L), respectively. CON (control), empty vector; shCON, shRNA control. (**d**) The expression levels of epithelial and mesenchymal markers in the indicated cells were analysed using immunoblotting. (**e**,**f**) DOT1L-overexpressing MCF10A and its control cells were treated with 100 pM TGF-β1 for 48 h, and subjected to immunoblotting for analysis of DOT1L expression and activity (**e**). The invasion (upper) and migration (lower) by the indicated cells were analysed and quantified (**f**). * and ^†^*P*<0.05 compared with CON/vehicle and CON/TGF-β, respectively (Student's *t*-test). (**g**) Analysis of invasion (left) and migration (right) by Tet-inducible DOTL-knockdown MDA-MB-231 cells. *P<0.05 versus shCON (Student's *t*-test). The data in **f,g** represent the means±s.d. of triplicate assays. (**h**) Effect of DOT1L on lung metastasis of breast cancer in xenografted mice. The *in vivo* experimental procedure was shown as a timeline (top). A representative images of histological analysis of lungs isolated from xenograft mice bearing Control (shCON) or Tet-inducible DOT1L-knockdown (shDOT1L)-MDA-MB-231 tumours (left). Arrowheads in the haematoxylin and eosin (H&E) image indicate lung metastatic nodules. To confirm DOT1L knockdown, immunohistochemical analysis of DOT1L expression was performed using lung tissues from the mice (bottom left). Data were quantified by counting the number of surface lung nodules (middle right) or assessing the incidence of lung metastasis in each group of mice (*n*=5; bottom right). Error bars indicate the means±s.e.m. *P<0.05 versus shCON (Student's *t*-test). Poisson distribution analysis was used for calculation of statistical significance of tumour incidence difference. Scale bars in **c**,**f**–**h**, 100 μm.

A recent study analysing a breast cancer genomic database implies that the *DOT1L* mRNA level is highly expressed in breast cancer and is especially associated with oestrogen receptor (ER)-negative breast cancer^10^. However, clinical evidence for role of DOT1L in breast cancer progression is still unclear. By using immunohistochemical analysis of DOT1L, we investigated the relationship of DOT1L with clinicopathological features in 182 human breast cancers. In our cohort, DOT1L was also associated with ER-α negativity (*P*=0.046, *χ*^2^-test), as well as progesterone receptor negativity (*P*=0.014, respectively, *χ*^2^-test) and triple-negative breast cancer (*P*=0.027, *χ*^2^-test), which are aggressive breast cancer subtypes (Supplementary Table 1). Furthermore, the high DOT1L expression level in the ER-negative invasive ductal carcinoma subtype was associated with significantly worse overall survival (*P*=0.042, Gehan–Breslow–Wilcoxon tests; hazard ratio, 2.624) but no statistically significant difference in recurrence (disease-free survival; *P*=0.184; Fig. 1a and Supplementary Table 2). DOT1L expression was not associated with survival in 182 patients and a subset of ER-positive breast cancers (Supplementary Table 2). We further found that higher DOT1L expression was significantly correlated with lymph node metastasis (*P*=0.003, *χ*^2^-test) and lymphatic invasion (*P*<0.001, *χ*^2^-test; Fig. 1b and Supplementary Table 1). Since Zhang *et al.*^10^ also showed that DOT1L inhibitors suppress breast cancer invasion *in vitro*, we next examined whether DOT1L is involved in regulation of EMT, an initiation step for tumour invasion and metastasis. In DOT1L-overexpressing MCF10A cells, a morphological change of epithelial cells into mesenchymal-like cells, loss of epithelial marker E-cadherin expression and gain of the expression of mesenchymal markers, N-cadherin and Vimentin were induced (Fig. 1c,d and Supplementary Fig. 1a,b). Moreover, treatment with DOT1L siRNA reversed the changes in the EMT marker expression in the DOT1L-overexpressing MCF10A cells, but not in the siRNA-resistant DOT1L mutant-expressing cells (Supplementary Fig. 1c). Consistently, tetracycline (Tet)-inducible DOT1L short hairpin RNA (shRNA) expression for conditional DOT1L knockdown in mesenchymal-like MDA-MB-231 cells, which have moderate DOT1L expression, led to a reversible morphological change, which suggested that the MET was accompanied by induction of E-cadherin and downregulation of mesenchymal markers (Fig. 1c,d). Transient DOT1L knockdown in MDA-MB-231 cells using multiple siRNAs also led to upregulation of E-cadherin (Supplementary Fig. 1d). Furthermore, DOT1L overexpression alone was sufficient for increasing the migration and invasion abilities of non-invasive MCF10A cells regardless of the treatment with transforming growth factor (TGF)-β, an EMT inducer, or TGF-β inhibitor SB 431542 (Fig. 1e,f and Supplementary Fig. 2a,b). This effect was reversed by inhibition of DOT1L enzymatic activity with EPZ004777 treatment (Supplementary Fig. 2a,b). In highly invasive and metastatic MDA-MB-231 cells, Tet-inducible DOT1L depletion inhibited these abilities (Fig. 1g). Similar results were shown in DOT1L-overexpressing T47D cells and DOT1L knockdown SK-BR-3 cells (Fig. 1c and Supplementary Fig. 3a–c). Furthermore, orthotopic xenograft mice with Tet-inducible DOT1L-knockdown MDA-MB-231 cells displayed significantly inhibited tumour metastasis in terms of the incidence of lung metastasis (four of five in controls versus one of five in DOT1L-knockdown cells) and number of nodules on the lung surface (Fig. 1h). The morphological analysis indicated that the lungs of control mice had numerous variable-sized infiltrative nodules composed of atypical cells, while the lungs of xenograft mice with DOT1L-knockdown cells showed well-preserved bronchiolar and alveolar structures without evidence of metastatic carcinoma (Fig. 1h, left). Collectively, our clinical, *in vitro* and *in vivo* evidence indicated that DOT1L is a marker of aggressive phenotype in human breast cancer that is associated with a worse clinical outcome in ER-negative breast cancer and advanced tumour progression related with EMT, invasion and metastasis.

### DOT1L induces breast tumorigenesis by enhancing CSC activity

**Figure 2:**
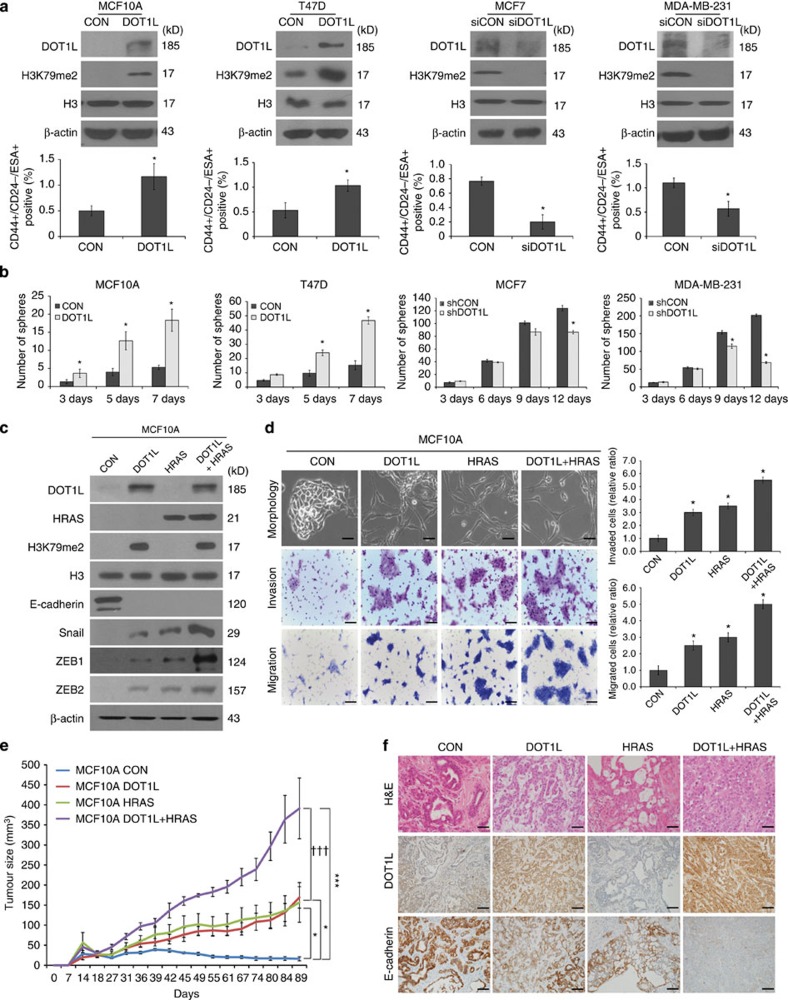
DOT1L is required for malignant transformation of breast epithelial cells and CSC-induced tumour initiation. (**a**) Lysates from stable DOT1L-expressing MCF10A and T47D cells and DOT1L siRNA (#1)-transfected MCF7 and MDA-MB-231 cells were subjected to immunoblotting for analysis of DOT1L and H3K79me2 expression (upper). The percentages of CD44^+^/CD24^−^/ESA^+^ breast CSC cells in the indicated cell lines were measured using flow cytometry (lower). siCON, control siRNA; siDOT1L, DOT1L siRNA. *P<0.05 versus controls (Student's *t*-test). (**b**) Tumoursphere formation assay was performed to evaluate self-renewal of CSCs in stable DOT1L-overexpressing and Tet-inducible DOT1L shRNA-expressing cells. shCON, pLKO-Tet control shRNA; shDOT1L, pLKO-Tet-DOT1L shRNA. *P<0.05 versus controls (Student's *t*-test). (**c**) Cell lines were generated by sequentially infecting MCF10A wild-type cells with activated HRAS and/or pLVX-DOT1L. Lysates from the indicated cells were then subjected to immunoblotting. (**d**) Morphological changes in the indicated cells were evaluated by microscopy (upper left). Images of invasion and migration assays in the indicated cells (middle and lower, left). Bars indicate measurements of invasion and migration abilities (right). *P<0.05 versus CON (Student's *t*-test). Results in (**a**,**b**,**d**) are shown as means±s.d. of experiments in triplicate. (**e**) The indicated stable MCF10A cells were orthotopically xenografted into NOD/SCID mice and tumour formation was monitored for 3 months. Representative tumour growth curves are shown. Error bars indicate the means±s.e.m. (*n*=5 mice). *P* values for multiple comparisons were calculated via repeated measures analysis of variance (RM ANOVA) followed by LSD *post hoc* test. *P=0.011 and 0.023, CON versus DOT1L and HRAS, respectively; ***P<0.001, CON versus DOT1L+HRAS; ^†††^*P*<0.001, DOT1L versus DOT1L+HRAS. (**f**) Representative images of H&E staining and immunohistochemistry for DOT1L and E-cadherin in the xenograft tumours from the indicated groups of mice. Scale bars in **d**,**f**, 100 μm.

Since the EMT phenomenon in CSCs has emerged as an important feature^11^,^12^, we next examined whether the increased EMT caused by DOT1L affects CSC-like phenotype in breast epithelial and cancer cells. By screening for DOT1L expression in several breast normal, immortalized and cancer cells, we found that DOT1L is upregulated in various breast cancer cells, especially in CD44^+^/CD24^−^/ESA^+^ breast CSC-like cell population, compared with non-transformed cells and non-CSCs, respectively (Supplementary Fig. 4a,b). In these cell lines, on the other hand, there was no significant correlation between DOT1L, H3K79me2 and E-cadherin expression (Supplementary Fig. 4a). Furthermore, consistent with a previous result suggesting decreased CSC self-renewal by DOT1L inhibitor^10^, lentiviral DOT1L overexpression in MCF10A and T47D cells, which have low DOT1L expression, expanded the CD44^+^/CD24^−^/ESA^+^ cell population and increased tumoursphere formation (Fig. 2a,b), whereas siRNA-mediated DOT1L knockdown in MDA-MB-231 and MCF7 cells, which express a high level of DOT1L, decreased the number of CSCs and tumourspheres (Fig. 2a,b). The inhibitory effect of DOT1L on CSC population was also confirmed using multiple DOT1L siRNAs in MDA-MB-231 and MCF7 cells (Supplementary Fig. 5). Furthermore, tumour xenograft with DOT1L-overexpressing T47D cells or Tet-inducible DOT1L shRNA-infected MDA-MB-231 cells in non-obese diabetic/severe combined immunodeficient (NOD/SCID) mice showed that DOT1L expression is required for tumour-initiating ability *in vivo* (Table 1). DOT1L also accelerated *in vitro* and *in vivo* tumour growth as assessed by soft-agar colony-forming assay and xenograft (Supplementary Fig. 6a–c). Together, these results indicate that DOT1L plays an important role in breast tumour initiation by enhancing the self-renewal and tumorigenic ability of CSCs. To confirm our finding that DOT1L is critical for regulation of CSCs and EMT, we investigated whether DOT1L could confer invasive and tumorigenic properties on MCF10A non-transformed but immortalized breast epithelial cells. Several oncogenic proteins, such as BMI-1 and LBX1, have been shown to cooperate with HRAS in promoting the EMT, metastasis and initiation of tumorigenesis^25^,^26^. Therefore, we also compared the effect of either DOT1L or HRAS alone and the combination of DOT1L and HRAS on the EMT and tumorigenesis by generating MCF10A cells co-expressing DOT1L and an activated form of HRAS (DOT1L+HRAS). In these cells, loss of E-cadherin and upregulation of Snail, ZEB1 and ZEB2 EMT-TFs leading to EMT morphological change and acceleration of migration and invasion were increased by either DOT1L or HRAS overexpression (Fig. 2c,d). These effects were further promoted when DOT1L and HRAS are co-expressed (Fig. 2c,d), and combinational inhibition of DOT1L and HRAS synergistically abolished the cell migration and invasion in these cells (lanes 10 versus 13; Supplementary Fig. 7a,b). However, DOT1L and HRAS independently functioned to promote EMT, since HRAS inhibition in MCF10A cells expressing DOT1L alone (lanes 2 versus 4) or DOT1L inhibition in MCF10A cells expressing HRAS alone (lanes 6 versus 7) could not remarkably suppress the migration and invasion abilities (Supplementary Fig. 7a,b). In addition, DOT1L and HRAS did not affect their expression of each other (Fig. 2c). We further investigated their abilities in generating tumours following orthotopic inoculation of MCF10A cells into NOD/SCID mice. Marked tumour formation in xenograft mice injected with either DOT1L- or HRAS-overexpressing cells was observed at 3 months following injection (Fig. 2e). Furthermore, mice injected with DOT1L+HRAS MCF10A cells exhibited more prominent tumour growth. Notably, histological analysis showed that DOT1L induced more poorly differentiated invasive ductal carcinomas compared with those induced by HRAS, and co-expression of DOT1L and HRAS resulted in more aggressive, high-grade invasive tumours with low E-cadherin expression (Fig. 2f), implying the additive effect of DOT1L and HRAS on breast tumorigenesis. Taken together, these results suggested that DOT1L is able to induce malignant transformation of breast epithelial cells, regardless of cooperation with HRAS, as a regulator of tumour-initiating ability of breast CSC.

### DOT1L expands CSCs via increasing EMT-TFs

**Figure 3:**
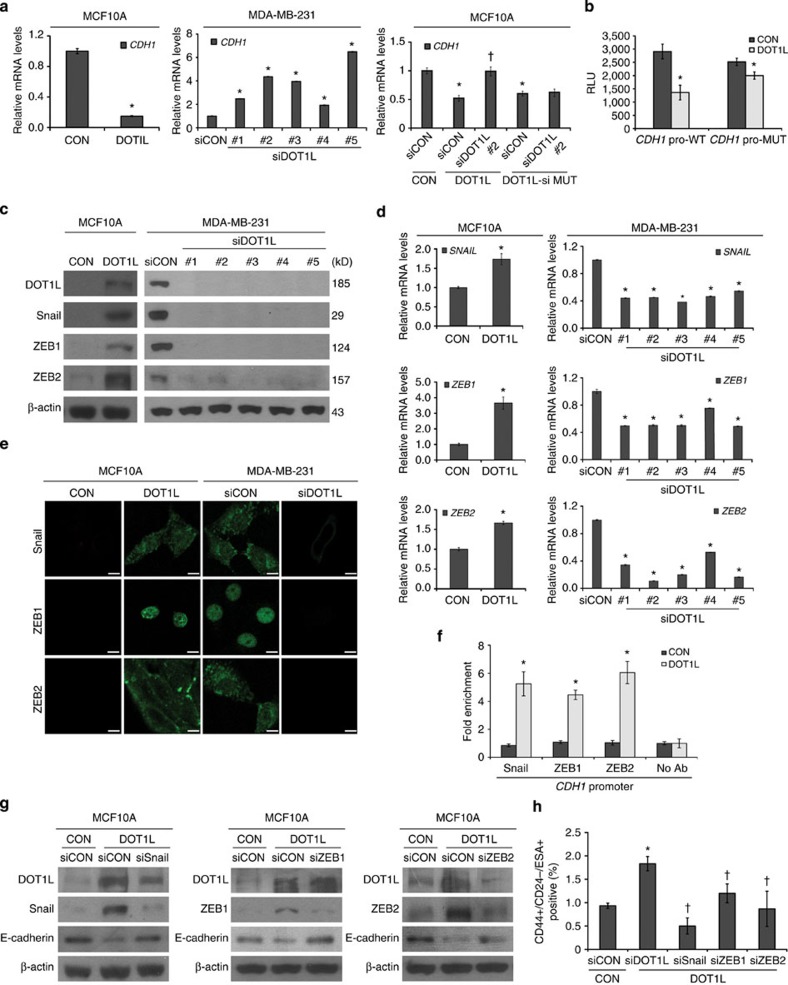
DOT1L increases Snail, ZEB1 and ZEB2 expression to repress E-cadherin expression and breast CSC property. (**a**) *CDH1* mRNA levels in the indicated cell lines were analysed using qRT–PCR. For transient knockdown of DOT1L, five different siRNAs against DOT1L (siDOT1L, #1–5) or siCON were transfected into MDA-MB-231 cells (middle). MCF10A cells expressing DOT1L wild-type (DOT1L) or DOT1L siRNA (#2)-resistant mutant (DOT1L-si MUT) were transfected with DOT1L siRNA #2 or control siRNA for 48 h, and subjected to qRT–PCR for analysis of *CDH1* expression (right). *P<0.05 versus controls (CON, siCON, CON/siCON) by Student's *t*-test. (**b**) Effect of DOT1L on *CDH1* promoter activity. Cells were transfected with luciferase constructs of wild-type (*CDH1* pro-WT) or E-box-mutant (*CDH1* pro-MUT) *CDH1* promoters for 24 h and the luciferase activity was measured. RLU, relative light units. *P<0.05 versus CON (Student's *t*-test). (**c**–**e**) Effects of DOT1L on the expression of *CDH1* transcriptional regulators were examined using immunoblotting (**c**), qRT–PCR (**d**) and immunofluorescence staining (**e**). *P<0.05 versus CON or siCON (Student's *t*-test). Scale bars in **e**, 100 μm. (**f**) Binding by EMT-TFs to the *CDH1* promoter region was analysed using ChIP–qPCR. *P<0.05 versus CON (Student's *t*-test). (**g**,**h**) Effect of EMT-TFs on DOT1L-induced cancer stemness. Cells were transfected with siRNAs against Snail, ZEB1 and ZEB2, and the CSC population was measured using flow cytometry. The knockdown of EMT-TFs was confirmed by immunoblotting (**g**). The FACS results were quantified and shown as a bar graph (**h**). * and ^†^*P*<0.05 versus CON/siCON and DOT1L/siCON, respectively (Student's *t*-test). Error bars in **a**,**b**,**d**,**f**,**h** indicate the means±s.d. of experiments in triplicate.

To further clarify the role of DOT1L in EMT and CSC, we investigated the molecular mechanism underlying the reduction by DOT1L of E-cadherin expression. On the basis of a previous finding showing DOT1L inhibitor-induced E-cadherin mRNA expression^10^, we confirmed that DOT1L regulates E-cadherin at the transcriptional level, as the mRNA level of the *CDH1* gene (encoding E-cadherin) was decreased by DOT1L overexpression in MCF10A cells (left) and increased by siRNA-mediated DOT1L knockdown in MDA-MB-231 cells (middle; Fig. 3a). Consistently, transient DOT1L knockdown rescued the repression of *CDH1* in DOT1L-overexpressing MCF10A cells, but not in DOT1L siRNA-resistant mutant-expressing cells (Fig. 3a, right). Furthermore, DOT1L repressed *CDH1* promoter activity and this effect was abolished by mutation of the E-box site within its promoter (Fig. 3b), indicating the importance of the E-box motif in DOT1L regulation of *CDH1*. Several EMT-TFs including Snail, ZEB1 and ZEB2 repress E-cadherin transcription by directly binding to the E-box motif within the *CDH1* promoter^13^. Consistent with the results in DOT1L-overexpressing MCF10A cells (Fig. 2c), the protein and mRNA levels of these EMT-TFs were positively regulated by DOT1L in both MCF10A and MDA-MB-231 cells, as assessed by immunoblotting, immunofluorescence staining and quantitative real-time PCR (qRT–PCR; Fig. 3c–e). This effect was abolished in the siRNA-resistant DOT1L mutant-expressing MCF10A cells (Supplementary Fig. 8). By performing chromatin immunoprecipitation–quantitative PCR (ChIP–qPCR) analysis, we further confirmed that DOT1L enhances direct binding of EMT-TFs on the *CDH1* promoter (Fig. 3f). Moreover, transient knockdown of Snail, ZEB1 and ZEB2 using their siRNAs recovered E-cadherin expression in DOT1L-overexpressing MCF10A cells (Fig. 3g). Thus, these results suggest that DOT1L represses the E-cadherin transcription through EMT-TF upregulation. Unexpectedly, DOT1L expression was also inhibited by Snail and ZEB2 knockdown (Fig. 3g), suggesting the possible positive feedback between these molecules. Because Snail, ZEB1 and ZEB2 modulate CSC activity^11^,^21^, we further explored whether DOT1L-induced CSC-like properties is mediated by these EMT-TFs. The transient knockdown of Snail, ZEB1 and ZEB2 halted the stem cell expansion in DOT1L-overexpressing MCF10A cells (Fig. 3h). Collectively, these results indicated that DOT1L-induced EMT-TF expression is critical for both enhancing EMT and CSC-like properties.

### DOT1L epigenetically derepresses EMT-TFs with c-Myc and p300

**Figure 4:**
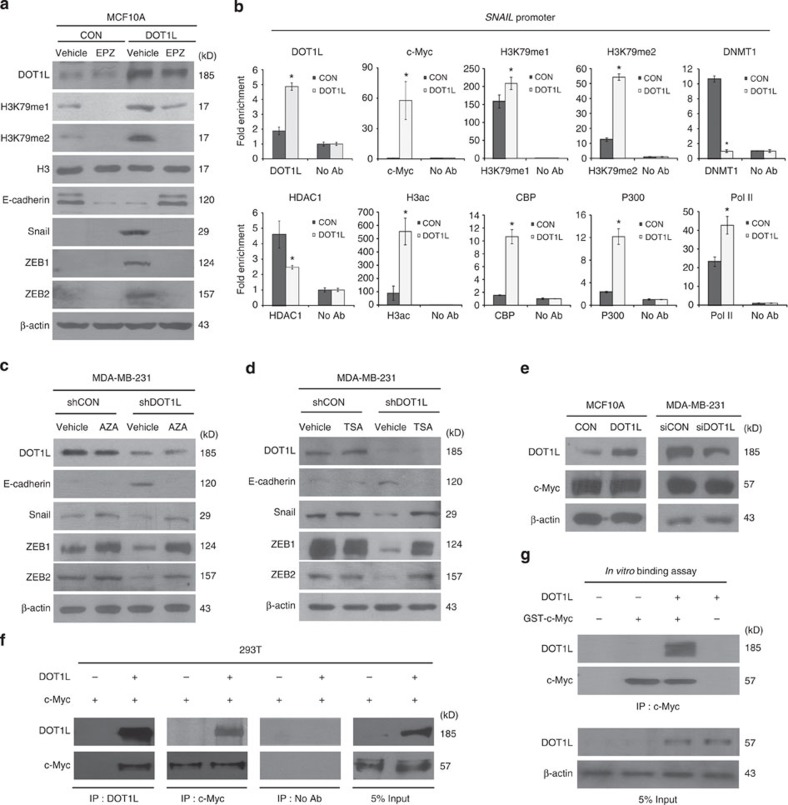
DOT1L epigenetically enhances EMT-TF expression in cooperation with histone acetyltransferases and c-Myc in the EMT-TF promoters. (**a**) Cells were treated with 1 μM EPZ004777 (EPZ) for 48 h and subjected to immunoblotting for analysis of Snail, ZEB1 and ZEB2 expression after inhibition of DOT1L enzymatic activity. (**b**) ChIP–qPCR analysis showing the amount of indicated proteins recruited to the promoter regions of Snail, ZEB1 and ZEB2. Data are presented as the means±s.d (n=3). *P<0.05 versus CON (Student's *t*-test). (**c**,**d**) The expression levels of EMT markers and transcriptional regulators were measured using immunoblotting after treatment of DOT1L-knockdown MDA-MB-231 cells with 5 μM 5'-Aza-2'-deoxycytidine (AZA) for 72 h and 100 ng ml^−1^ Trichostatin A (TSA) for 12 h. (**e**) The effect of DOT1L on c-Myc expression was analysed using immunoblotting. (**f**) Lysates from 293T cells transfected with DOT1L and/or c-Myc cDNA constructs were immunoprecipitated using the indicated antibodies and subjected to immunoblotting. (**g**) *In vitro* pull-down assay was performed to confirm the interaction between DOT1L and c-Myc. GST-c-Myc was used to pull down DOT1L from DOT1L-transfected 293T cell lysates. c-Myc-bound DOT1L was detected by indicated antibodies. Expression levels of DOT1L in 5% input were shown as indicated. Data are representative of three independent experiments.

We next elucidated the molecular mechanism underlying the regulation by DOT1L of the EMT-TF expression. Consistent with a previous study showing the enrichment of H3K79me2 in several mesenchymal regulator genes including *SNAIL*, *ZEB1* and *ZEB2* in human fibroblasts^24^, inhibition of DOT1L enzymatic activity with EPZ004777 treatment reversed DOT1L-induced EMT-TF expression in MCF10A cells (Fig. 4a). Moreover, ChIP–qPCR analysis showed that DOT1L increases the EMT-TF expression at the transcriptional level by direct recruitment and subsequent enrichment of H3K79me1 and H3K79me2 levels in the proximal promoter region of these genes (Fig. 4b and Supplementary Fig. 9a,b). In addition, consistent with a previous report showing the cooperation between DOT1L and RNA pol II in an actively transcribed genomic region^2^, the RNA pol II was enriched in the EMT-TF promoter regions together with DOT1L and H3K79 methylation. We further clarified the possibility of crosstalk between DOT1L-mediated H3K79me and several epigenetic modifications such as DNA methylation and histone acetylation in these promoters. The recruitment of histone acetyltransferase CBP/p300 and consistent enrichment of H3 acetylation (H3ac) in these promoters were observed, while HDAC1 was dissociated from the region in DOT1L-overexpressing cells (Fig. 4b and Supplementary Fig. 9a,b). Moreover, in MDA-MB-231 cells, inhibition of DNMT or HDAC activity using 5′-aza-2′-deoxycytidine or trichostatin A induced re-expression of EMT-TFs in DOT1L-knockdown MDA-MB-231 cells (Fig. 4c,d). We also analysed the c-Myc status in these promoters in DOT1L-overexpressing cells, as these promoter regions commonly contain putative E-box motifs for c-Myc binding and *SNAIL* is regulated by the c-Myc and smad2/3 complex^27^. Although DOT1L did not change the total level of c-Myc expression (Fig. 4e), c-Myc was recruited to the DOT1L-occupied region of EMT-TF promoters (Fig. 4b and Supplementary Fig. 9a,b). Furthermore, *in vitro* and *in vivo* protein-binding assays showed that DOT1L directly interacts with c-Myc (Fig. 4f,g), implying the formation of the DOT1L-c-Myc complex in regulation of EMT-TF transcription. Together, these data indicated that DOT1L enhances EMT-TF expression by increasing K79 methylation and acetylation towards histone H3 and c-Myc recruitment in the EMT-TF promoters.

### c-Myc is prerequisite for DOT1L-induced EMT-TF regulation

**Figure 5:**
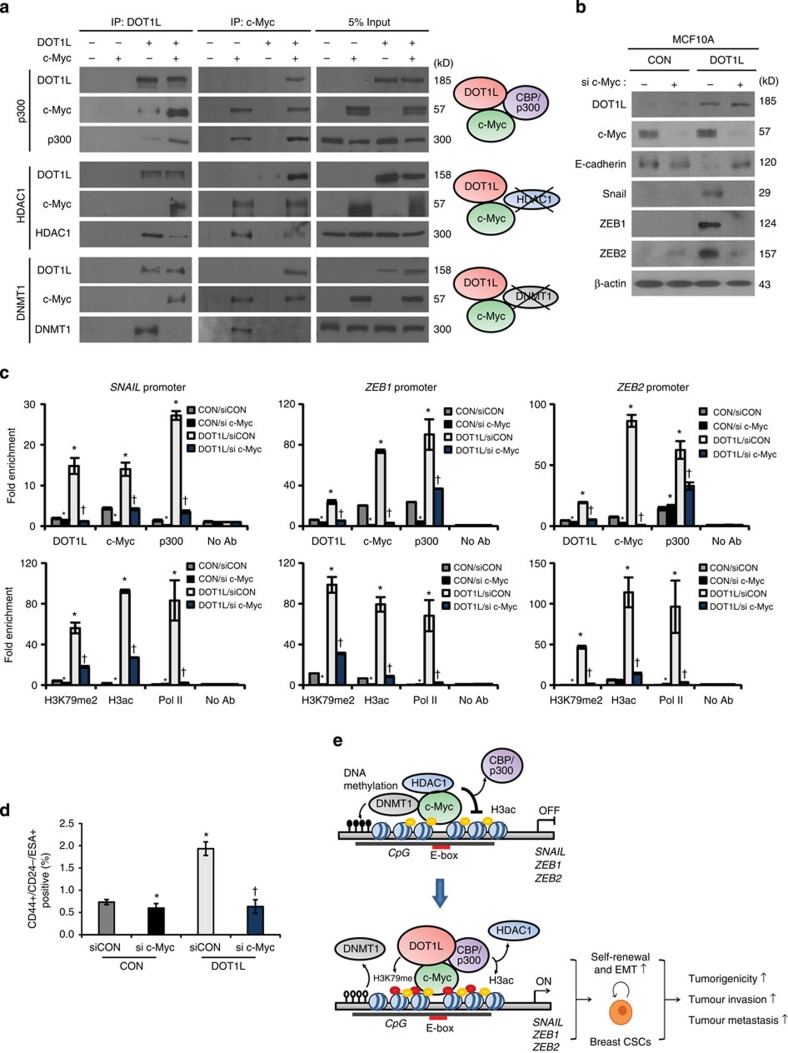
c-Myc is required for formation of DOT1L-containing transcriptional active complex and recognition of DOT1L on EMT-TFs in regulation of breast CSC activity. (**a**) To confirm the binding of DOT1L or c-Myc with the indicated proteins, co-immunoprecipitation assays were performed using lysates from 293T cells transfected with the indicated constructs. (**b**) To investigate the effect of c-Myc on DOT1L-induced EMT-TF expression, lysates from CON or DOT1L-overexpressing MCF10A cells transfected with control or c-Myc siRNA (si c-Myc) were analysed by immunoblotting. (**c**) Dependency of EMT-TF regulation by DOT1L on c-Myc was confirmed using ChIP–qPCR assay in c-Myc siRNA-transfected MCF10A cells expressing DOT1L or its control cells. (**d**) The CSC population after c-Myc siRNA transfection was measured by FACS. Results in **c**,**d** are shown as means±s.d. of experiments in triplicate. * and ^†^*P*<0.05 versus CON/siCON and DOT1L/siCON, respectively (Student's *t*-test). (**e**) A proposed model for the regulation by DOT1L of EMT-TFs. In the absence of DOT1L, c-Myc forms a repressive complex with DNMTs and HDACs in the E-box motif of the promoter regions of Snail, ZEB1 and ZEB2 EMT-TFs, which leads to DNA methylation at the CpG island and histone deacetylation within the promoters. When DOT1L is overexpressed, c-Myc interacts directly with the DOT1L complex containing HATs and recruits them to the promoter region of EMT-TFs. This leads to dissociation of DNMTs and HDACs from the promoters and enrichment of H3K79me and H3 acetylation in the region for activation of EMT-TF transcription. The enhanced expression of EMT-TFs induces EMT-associated CSC and metastasis.

Because c-Myc was a common regulatory factor for DOT1L-induced *SNAIL*, *ZEB1* and *ZEB2* transcription (Fig. 4b and Supplementary Fig. 9a,b), we further examined the relationship between c-Myc, DOT1L and histone acetylation and DNA methylation modifiers in EMT-TF regulation. Interestingly, DOT1L bound preferentially to p300 rather than to HDAC1 and DNMT1 under c-Myc overexpression conditions (Fig. 5a). Similarly, the binding of c-Myc to p300 was increased by DOT1L overexpression, even though c-Myc could bind to both the HDAC1 and DNMT1 transcriptional repressive complex and p300 active complex. These data implied that DOT1L forms a transcriptionally active complex with c-Myc and p300. Because the different binding of epigenetic factors with DOT1L protein was dependent on c-Myc expression, we next examined whether c-Myc was required for DOT1L-mediated epigenetic alteration and subsequent EMT-TF activation. The knockdown of c-Myc using siRNA blocked the increased EMT-TF expression caused by DOT1L overexpression in MCF10A cells (Fig. 5b). Furthermore, the binding of DOT1L and p300/CBP to the promoter region of EMT-TFs and dissociation of HDAC1 and DNMT1 from the promoters were dependent on the c-Myc status, as confirmed using ChIP assay after inhibition of c-Myc expression (Fig. 5c and Supplementary Fig. 10). In the control MCF10A cells having very low DOT1L expression, there were no detectable changes in the expression of EMT-TFs despite of c-Myc knockdown as assessed by immunoblotting (Fig. 5b), while DNMT1 and HDAC1 were recruited more firmly to the EMT-TF promoters by c-Myc depletion (Supplementary Fig. 10), indicating the requirement of DOT1L for c-Myc-mediated EMT-TFs upregulation. We also confirmed that the increased CSC population caused by DOT1L was abolished by c-Myc knockdown using siRNA (Fig. 5d), implying that c-Myc could affect the DOT1L-induced breast CSC activation. Collectively, DOT1L is recruited to EMT-TF promoters such as SNAIL, ZEB1 and ZEB2 together with c-Myc and CBP/p300 co-activator complex for epigenetic transactivation of these EMT-TFs with enrichment of H3K79me and H3ac. These results also suggest that c-Myc is required for recognition by DOT1L of target chromatin and formation of a transcriptionally active complex with DOT1L-c-Myc-p300 for epigenetic regulation of EMT-TF-associated cancer stemness and tumour progression.

## Discussion

In this study, we demonstrated that cooperation of DOT1L with c-Myc-p300 is important for regulation of both the EMT and CSC in breast cancer by epigenetic activation of EMT-TFs, providing a novel mechanism of epigenetic regulation of DOT1L-mediated transcription of EMT enhancers (Fig. 5e). For transactivation of the EMT regulators, DOT1L is recruited to the E-box regions of *SNAIL*, *ZEB1* and *ZEB2* promoters together with c-Myc and CBP/p300 co-activator complex, and enhances H3K79me- and H3ac-induced epigenetic derepression of EMT-TFs. Moreover, DOT1L leads to dissociation of HDAC1 and DNMT1 proteins from promoters, which inhibits HDAC activity and DNA methylation. This process results in the acquisition of EMT properties and enhancement of invasive and metastatic abilities and CSC properties. Therefore, our data suggest that DOT1L-c-Myc-p300 complex-mediated epigenetic control of EMT-TFs is important for regulation of the EMT and EMT-associated CSCs in breast tumour initiation and progression.

Our findings are first to demonstrate that DOT1L and c-Myc cooperation provides an important molecular link between the EMT and CSCs. Growing evidence has shown the functional association of EMT and CSCs. EMT and metastasis are the main characteristics of CSCs^11^,^12^. EMT induces adult stem-like epithelial cells that have self-renewal capabilities and chemoresistant properties. The self-renewal of CSCs is required for metastatic colonization of distant organs^12^,^15^. Furthermore, many EMT-TFs and CSC modulators are closely associated with the regulation of this phenomenon. For instance, ZEB1 represses several miRNAs targeting a CSC marker Bmi-1 to maintain CSC properties^21^. The self-renewal regulatory Wnt/β-catenin signalling could induce Twist and Slug expression to enhance EMT and metastasis^15^. In EMT progression, c-Myc, a well-known activator of self-renewal of normal and malignant stem cells, also has been known to enhance Snail transcription via direct or indirect pathway, as a transcription factor or ERK-dependent GSK-3β activation^18^,^27^. We here further demonstrate that that c-Myc is a common transcription factor of EMT-TFs including Snail, ZEB1 and ZEB2 via direct association with their promoter region containing E-box motifs, suggesting the critical role of c-Myc in regulation of EMT. Furthermore, our novel findings showed that these EMT-TFs are upregulated by DOT1L-induced epigenetic modifications, including histone methylation and acetylation and DNA methylation, in collaboration with the c-Myc, during the EMT and CSC processes. Together with previous findings providing the cooperation of epigenetic modifiers and EMT-TFs in epigenetic gene silencing of E-cadherin^22^,^23^, these data could suggest that EMT-TFs, upstream signalling of E-cadherin, are also tightly controlled by an epigenetic and transcriptional co-regulatory complex. Collectively, we suggest that DOT1L and c-Myc-p300 complex cooperation is critical for tumour initiation and progression linking the EMT and CSC phenomenon as a crucial mechanism of EMT-TF induction.

We also provide the novel molecular function of DOT1L in MLL complex-independent transcriptional regulatory programme by showing that DOT1L forms a transcriptional activation complex with c-Myc and p300 acetyltransferase to increase the expression of EMT regulators. DOT1L has been known to contribute to the initiation and maintenance of active transcription in the genome by associating with multiple complexes besides its own enzymatic activity towards H3K79 (refs 1, 2, 3). In the MLL fusion protein complex, DOT1L is involved in RNA pol II-mediated transcriptional elongation^6^,^7^,^8^. A recent study also showed that DOT1L binds directly to actively transcribing RNA pol II to facilitate gene transcription^2^. In this study, we identified c-Myc as a critical factor for DOT1L-associated transcriptional activation as a novel interacting partner of DOT1L. Furthermore, our data showed the DOT1L and c-Myc association with RNA pol II in the EMT-TF promoter region, supporting the evidence that the DOT1L-c-Myc complex functions as a transcriptional activator. Typically, c-Myc binds to the E-box within the promoter region of target genes together with its common binding partners, such as MAX. Furthermore, a genome-wide study showed that c-Myc binds to chromatin regions marked with methylated H3K4 and H3K79me as well as acetylated H3 (ref. 28). Because our data showed c-Myc-dependent DOT1L recruitment to promoter regions of target genes, we suggest that DOT1L recognizes target chromatin through the DNA-binding ability of c-Myc. c-Myc controls both the activation and repression of gene expression by collaborating with multiple transcriptional and epigenetic modifiers. For instance, TRRAP, the first identified cofactor of c-Myc-induced transcription, recruits several histone acetyltransferases to c-Myc target genes for gene activation^29^. c-Myc can also recruit directly CBP and p300 acetyltransferases to target chromatin^30^,^31^. Conversely, c-Myc could associate with HDAC or DNMT proteins to form a transcriptional repressive complex^32^,^33^. Our data suggest that the c-Myc-dependent transcriptional switch is modulated by DOT1L, as in the presence of DOT1L c-Myc preferentially forms an active complex with p300 rather than a repressive complex containing HDAC1 and DNMT1. Although further studies are needed to more clearly evaluate the core components of DOT1L and c-Myc-containing transcription regulatory complex and their roles, these data possibly suggest that DOT1L may facilitate the formation of c-Myc-containing transcriptional active complex in competition with the original c-Myc-binding partners involving transcriptional repression in c-Myc-bound genomic regions. Taken together, our findings demonstrate that DOT1L is a main component of c-Myc-dependent transcriptional and epigenetic regulatory programme, suggesting the crucial role of DOT1L and c-Myc cooperation in facilitation of target gene activation.

It is notable that our findings provide the clear clinical, *in vitro* and *in vivo* evidence for a solely oncogenic role of DOT1L regardless of MLL fusion partners, in malignant transformation, invasion and metastasis of solid tumour. Numerous studies have proposed DOT1L as a crucial oncogene and potential therapeutic target in human leukaemia, while only two studies have reported a role for DOT1L in accelerating cell proliferation in some solid tumours, and prostate and lung cancers^4^,^34^. During leukaemogenesis, DOT1L was identified as a binding protein of frequent translocation partners of MLL, such as AF10, ENL, ELL, AF4 and AF9 (refs 6, 35, 36, 37). Furthermore, these studies showed the importance of DOT1L enzymatic activity towards H3K79 in the regulation of the target genes of MLL fusion partners. A recent study reports that pharmacological inhibition of H3K79 methylation is also effective on breast cancer cell proliferation and migration and invasion *in vitro* assay^10^. Herein, our *in vitro* and *in vivo* data demonstrated that DOT1L conferred tumorigenic potential on MCF10A non-transformed human breast epithelial cells in either the presence or absence of the RAS oncogene. Moreover, as an enhancer of EMT and CSC, DOT1L contributed to breast tumour initiation and EMT-mediated invasion and metastasis. These data suggest DOT1L to be a powerful oncogene driving breast tumorigenesis in tumour initiation and progression. Recently, several potent catalytic inhibitors of DOT1L that cause tumour regression in MLL-rearranged leukaemia have been discovered and are undergoing clinical trials^9^,^38^. Because the DOT1L enzymatic activity of H3K79me was also important for CSC, EMT and metastasis in our study, these DOT1L-target drugs might also be useful for breast cancer therapy. Our clinical data show the association of high DOT1L expression with invasion and metastasis of human breast cancers, as well as the poorer survival rate in an aggressive subset of ER-negative breast cancers. Although the DOT1L did not affect the survival of ER-positive breast cancer patients who have a favourable outcome following anti-oestrogen therapy, our clinical data support the notion that DOT1L could be a therapeutic target in more aggressive ER-negative and metastatic human breast cancers. Further extensive meta-analyses of clinical trials are necessary. On the basis of our results, we suggest that DOT1L is a critical indicator of breast cancer aggressiveness that induces breast cancer initiation and progression, invasion and metastasis because of its enhancement function of the CSC and EMT.

In conclusion, we suggest DOT1L to be critical for regulation of EMT-induced tumour initiation and progression in breast cancer as an important epigenetic modifier of EMT-TFs by collaborating with the c-Myc-p300 complex. Therefore, DOT1L could be an important oncogene and a novel target in aggressive breast cancer therapy.

## Methods

### Patients and surgical specimens

In this study, consecutive primary human breast cancers obtained from 182 breast cancer patients at the Hanyang University Hospital (Seoul, Korea) from January 2000 to December 2005 under the approval of Institutional Review Boards of Hanyang University Hospital were used. Informed consent was provided by all patients enrolled in the study. Histopathological and clinical data were obtained from pathology reports and medical records, including various conventional clinicopathological parameters and information concerning adjuvant chemotherapy, radiotherapy and endocrine treatment as well as follow-up data.

### Tissue microarray and immunohistochemical staining

To define the diagnostic area, haematoxylin and eosin-stained sections from each paraffin-embedded, formalin-fixed block were used. One representative 0.6-mm core was obtained from each case and inserted in a grid pattern into a recipient paraffin block using a tissue arrayer (Beecher Instruments, Silver Spring, MD, USA). Sections 4 μm in thickness were then cut from each tissue microarray (TMA) block and immunostained with antibodies to DOT1L (1:200, Abcam, ab64077, Cambridge, MA, USA) using the Bond-Max autostainer (Leica, Wetzlar, Germany). Each core was evaluated by estimating the percentage and intensity of tumour cells showing a nuclear staining pattern. Samples were considered positive if 30% or more of the tumour cells were immunostained.

### Cell culture and reagents

All cells were obtained from the American Type Culture Collection (Manassas, VA, USA). Human breast cancer cell lines were cultured in phenol red-free DMEM (Welgene, Daegu, Korea) supplemented with 10% fetal bovine serum at 37 °C in a humidified 5% CO_2_ incubator. MCF10A human breast epithelial cell lines were maintained in DMEM/F12 medium (Welgene) supplemented with 5% horse serum, 20 ng ml^−1^ epidermal growth factor (EGF), 10 mg ml^−1^ insulin and 0.5 mg ml^−1^ hydrocortisone. To activate the Tet-inducible system, cells were maintained in DMEM containing 10% Tet-free FBS (Welgene) and 2 μg ml^−1^ doxycycline (DOX; Clontech, Mountainview, CA, USA). TGF-β inhibitor SB 431542, 5′-Aza-2′-deoxycytidine and trichostatin A were obtained from Sigma-Aldrich (St Louis, MO, USA). DOT1L inhibitor EPZ004777 was purchased from Glixx Laboratories (Southborough, MA, USA). RAF kinase inhibitor Sorafenib was purchased from Santa Cruz Biotechnology (Santa Cruz, CA, USA). For transient knockdown of DOT1L, Snail, ZEB1, ZEB2 and c-Myc, non-targeting siRNA and siRNAs against these genes (DOT1L siRNA #1, 5′-CAGUGAUGGUGCUUCUCUU-3′, cat no. 1043628; DOT1L siRNA #3, 5′-GAAGUGGAUGAAAUGGUAU-3′, cat no. 1043632; DOT1L siRNA #4, 5′-GACCUGAUUCAAGCGCAGA-3′, cat no. 1043624; DOT1L siRNA #2, 5′-GCUGGAGCUGAGACUGAAG-3′; DOT1L siRNA #5, 5′-GCAUGCAGAAUACACAUUG-3′; c-Myc siRNA, 5′-GACAGUGUCAGAGUCCUGA-3′, cat no. 1100224; Snail siRNA, 5′-GACUGUGAGUAAUGGCUGU-3′, cat no. 1141908; ZEB1 siRNA, 5′-AGUCCUUUGAAGAUGACUA-3′, cat no. 1149356; ZEB2 siRNA, 5′-UCAGCAUGAACGUUACCUU-3′, cat no. 1165992) were purchased from Bioneer and transfected into cells for 48 h using Lipofectamine 2000 (Invitrogen, Carlsbad, CA, USA), as described by the manufacturer.

### Lentiviral overexpression and knockdown system

To establish DOT1L-overexpressing cell lines, human DOT1L cDNA kindly provided by Daeyoup Lee (KAIST, Daejeon, Korea) was subcloned into lentiviral pLVX-puro vector (Clontech). 293FT cells were then co-transfected with pLVX-DOT1L or its empty vector construct and packaging mix using Lipofectamine 2000 reagent (Invitrogen) to generate the lentiviral particles. After 48–72 h of transfection, the medium containing lentiviruses was harvested and transduced into target cells with 8 μg ml^−1^ Polybrene (Sigma-Aldrich). To generate the DOT1L shRNA-expressing pLKO-Tet-On vector, a pair of oligonucleotides encoding DOT1L shRNA (sense, 5′-CCGGCCCGAGAAGCTCAACAACTCTCGAGAGTTGTTGAGCTTCTCGGGTTTTT-3′ and antisense, 5′-AATTAAAAACCCGAGAAGCTCAACAACTCTCGAGAGTTGTTGAGCTTCTCGGG-3′) was annealed and subcloned into the pLKO-Tet-On vector (Sigma-Aldrich). The lentiviruses encoding Tet-inducible DOT1L shRNA were then generated and infected into cells as described above. For expression of shRNA against DOT1L, cells were treated with 2 μg ml^−1^ DOX for 48 h.

### Generation of DOT1L siRNA-resistant cell lines

To establish the DOT1L siRNA-resistant cell lines, the pCDH-DOT1L siRNA-resistant mutant construct against DOT1L siRNA #2 (5′-GCUGGAGCUGAGACUGAAG-3′) was kindly provided by Ja-Eun Kim (Kyung Hee University, Seoul, Korea). The DOT1L mutant cDNA was then subcloned into lentiviral pLVX-puro vector, and generation of the lentiviral overexpression was performed as described above.

### RT–PCR and qRT–PCR

Total RNA was extracted using TRIzol reagent (Invitrogen) as described by the manufacturer, and Reverse transcription–PCR (RT–PCR) was performed according to the instructions provided using the Access RT–PCR Systems (Promega, Madison, WI, USA). To quantify the RNA expression levels, qRT–PCR was performed on the Applied Biosystems 7300 Real-Time PCR system using SYBR Green dye (Applied Biosystems, Foster City, CA, USA) as described by the manufacturer, and data were normalized to expression of a control gene *GAPDH*. The following primers were used for qRT–PCR: *DOT1L*, 5′-CAAGTTCTCGCTGCCTCACT-3′ and 5′-GTCCTGAGGGCTCAGCTTC-3′; *CDH1*, 5′-TGCCCAGAAAATGAAAAAGG-3′ and 5′-GTGTATGTGGCAATGCGTTC-3′; *SNAIL*, 5′-AGCCTGGGTGCCCTCAAGATG-3′ and 5′-CTTGGTGCTTGTGGAGCAGGGAC-3′; *ZEB1*, 5′-GCACCTGAAGAGGACCAGAG-3′ and 5′-GTGTAACTGCACAGGGAGCA-3′; *ZEB2*, 5′-TTCCTGGGCTACGACCATAC-3′ and 5′-GCCTTGAGTGCTCGATAAGG-3′; *GAPDH*, 5′-GAAGGTGAAGGTCGGAGTC-3′ and 5′-GAAGATGGTGATGGGATTTC-3′; *18s rRNA*, 5′-GTAACCCGTTGAACCCCATT-3′ and 5′-CCATCCAATCGGTAGTAGCG-3′.

### Immunoblotting and immunoprecipitation

Cells were lysed with radioimmunoprecipitation assay buffer (50 mM Tris, pH 7.4, 150 mM NaCl, 1 mM EDTA, 1% NP-40, 0.25% sodium deoxycholate) supplemented with protease and phosphatase inhibitors, and were briefly sonicated. For immunoblotting, the cell lysates were separated by 6–12% SDS–SDS–PAGE and transferred to nitrocellulose membranes. The membranes were blocked with 5% non-fat dry milk, incubated with primary antibodies for overnight at 4 °C and subsequently reacted with horseradish peroxidase-conjugated secondary antibodies (sc-2004 and sc-2005, Santa Cruz Biotechnology) for 1 h at room temperature. Bands were visualized using the ECL detection system (GE Healthcare, Chalfont St Giles, UK). For immunoprecipitation, cell lysates (1–2 mg) were incubated with appropriate antibodies (1 μg) overnight at 4 °C. The immunoprecipitates were incubated with protein A or G-agarose (20 μl) for 2 h at 4 °C and washed with ice-cold PBS three times, and then Laemmli sample buffer was added to the immunoprecipitated pellets. The samples were heated at 95 °C for 5 min and then analysed using SDS–PAGE and immunoblotting as described above.

Antibodies specific for the following factors were used for immunoblotting at indicated dilution and/or for immunoprecipitation: DOT1L (1:2,000, ab64077), H3K79me2 (1:3,000, ab3594) and N-cadherin (1:2,000, ab12221) from Abcam; Histone H3 (1:3,000, 9715S) and c-Myc (1:1,000, 5605) from Cell Signaling Technology (Beverly, MA, USA); Vimentin (1:3,000, sc-32322), Snail (1:1,000, sc-28199), ZEB1 (1:2,000, sc-25388), ZEB2 (1:1,000, sc-48789), CBP (1:2,000, sc-369), DNMT1 (1:1,000, sc-20701), HDAC1 (1:2,000, sc-7872), HRAS (1:1,000, sc-29) and RNA pol II (sc-9001) from Santa Cruz Biotechnology; E-cadherin (1:1,000–1:5,000, 610181) from BD Biosciences (Palo Alto, CA, USA); H3ac (06–599), p300 (1:2,000, 05–257) and β-actin (1:10,000, MAB1501R) from Millipore (Billerica, MA, USA). Uncropped images of all blots are in Supplementary Fig. 11.

### *In vitro* pull-down assay

Glutathione S-transferase (GST)-recombinant GST-c-Myc proteins (Abnova, Taipei, Taiwan) were immunoprecipitated using anti-c-Myc antibody (1 μg, Cell Signaling Technology) and conjugated with protein A-agarose beads. Subsequently, the immortalized c-Myc proteins were reacted with cell lysates from 293T cells transfected with the pCI-neo empty vector or pCI-neo-DOT1L construct for pull-down assay. The amount of c-Myc-associated DOT1L protein was then detected by immunoblotting.

### Flow cytometry analyses

For analysis of breast CSC population, cells were stained with allophycocyanin-conjugated CD44 (1:200, 559942, BD Biosciences, San Jose, CA, USA), phycoerythrin (PE)-conjugated CD24 (1:200, 555428, BD Biosciences) and fluorescein isothiocyanate-conjugated epithelial surface antigen (ESA) (1:200, F0860, Dako, Carpenteria, CA, USA) antibodies at 4 °C for 15 min. The subpopulation of CD44^+^/CD24^−^/ESA^+^ cells was then measured using a FACS Canto 2 (Becton Dickinson, San Jose, CA, USA). Because of GFP interference of lentiviral DOT1L shRNA, transient DOT1L knockdown cells using siRNA were used for analysis of flow cytometry. To isolate CSC and non-CSC populations from breast cancer cell lines, cells were stained with the above antibodies, and the subpopulation of CD44^+^/CD24^−^/ESA^+^ cells and the other populations were sorted using a FACS Aria flow cytometer (BD Biosciences). Each sorted cell population was collected and total RNA was extracted for DOT1L RT–PCR. To measure E-cadherin expression using flow cytometry, permeabilized cells were stained with Alexa Fluor 488-conjugated E-cadherin antibody (1:50, 3199, Cell Signaling) at 4 °C for 30 min and the percentage of Alexa Fluor 488-positive cells was analysed using FACS Canto 2.

### Tumoursphere formation assay

MCF10A (5 × 10^3^ cells per well) and MDA-MB-231 (5 × 10^3^ cells per well) cells were cultured in DMEM-GlutaMAX medium (Invitrogen) supplemented with B27 (Invitrogen), 10 ng ml^−1^ basic fibroblast growth factor (Peprotech, Rocky Hill, NJ, USA), 10 ng ml^−1^ EGF and 4 ng ml^−1^ heparin (Sigma-Aldrich) in a six-well ultra-low attachment surface plate (Corning, Corning, NY, USA). MCF7 (1 × 10^4^ cells per well) cells and T47D (1 × 10^4^ cells per well) cells were each cultured in DMEM-GlutaMAX medium containing B27, 20 ng ml^−1^ basic fibroblast growth factor, 20 ng ml^−1^ EGF and 4 ng ml^−1^ heparin (Sigma-Aldrich) in a six-well ultra-low attachment surface plate. For Tet-inducible DOT1L knockdown, 2 μg ml^−1^ DOX was added to the medium at the time of plating. After 3, 5 and 7 days, tumoursphere formation was examined using an inverted microscope and the number of tumourspheres formed (>100 μm) was quantified.

### Soft-agar colony formation assay

To measure anchorage-independent cell growth, 1% agar in growth medium was coated on six-well plates. MCF10A and MDA-MB-231 cells (each 5 × 10^3^ cells per well), T47D (1 × 10^4^ cells per well) and MCF7 (3 × 10^4^ cells per well) cells were then plated in 0.4 and 0.3% agars on the bottom agar, respectively. For induction of DOT1L shRNA expression, MDA-MB-231 and MCF7 cells were treated with 2 μg ml^−1^ DOX every 3 days during the assay. After incubation for 15 days, colonies >100 μm in size were counted under a microscope.

### Immunofluorescence staining

Cells were seeded at a density of 8 × 10^4^ on a four-chamber slide glass overnight, washed and fixed with cold methanol for 5 min. After blocking with 3% BSA for 1 h, the cells were stained with anti-E-cadherin, N-cadherin, Vimentin, Snail, ZEB1 and ZEB2 (1:200 dilution) at 4 °C overnight and further incubated with anti-rabbit immunoglobulin/R-PE (1:400, P9795, Sigma) for 1 h and additionally stained with DAPI for 10 min to visualize the nucleus. A fluorescence microscope (Nikon, Tokyo, Japan) was used for detection of immunofluorescence.

### Luciferase reporter assay

The wild-type and E-box-mutant *CDH1* promoter constructs were purchased from Addgene, deposited by Eric Fearon (University of Michigan Medical School, Ann Arbor, MI, USA). Cells were seeded in a 12-well plate and transfected with the reporter constructs and a β-galactosidase expression vector for 24 h. A luciferase reporter assay was then performed using a Luciferase Assay System (Promega). The luciferase activity, expressed as relative light units, was normalized to β-galactosidase activity.

### ChIP–qPCR assays

ChIP assays were performed following the manufacturer's instructions provided with the kit (Upstate Biotechnology, Lake Placid, NY, USA). In brief, cells were crosslinked with 1% formaldehyde at room temperature for 10 min and the reaction was stopped by treatment with 0.125 M glycine. The cell pellets were resuspended in 200 μl of SDS lysis buffer and sonicated using a Bioruptor (Cosmo Bio Co. Ltd, Tokyo, Japan). The cell lysates were then immunoprecipitated with specific antibodies overnight at 4 °C and further incubated with salmon sperm DNA coupled to protein A-agarose (Millipore) for 2 h at 4 °C. The precipitates were washed, eluted and then reverse-crosslinked with 20 μl 5 M NaCl by incubating at 65 °C overnight. DNA fragments were precipitated from the eluate and dissolved in ddH_2_O. The enrichment of the ChIP signal was analysed using qPCR (signal/input ratio) as described above using the following specific primers: *CDH1* promoter (forward, 5′-AGGCTAGAGGGTCACCGCGTC-3′, reverse, 5′-GCTTTGCAGTTCCGACGCCAC-3′), *SNAIL* promoter (forward, 5′-GTACTTAAGGGAGTTGGCGG-3′, reverse, 5′-CCGATTCGCGCAGCAGTA-3′), *ZEB1* promoter (forward, 5′-GCACAGGGTACAGGGAGAAT-3′, reverse, 5′-GGTAAAGTTGGAGGCTCGGC-3′) and *ZEB2* promoter (forward, 5′-GAAGGGAGGGAGGTGGAATTT-3′, reverse, 5′-CGCCAAGTTTCTCTCTGGGAA-3′).

### Transwell migration and invasion assays

The cell migration and invasion abilities were evaluated as described previously^39^. Briefly, for the migration assay, cells were seeded in the upper chamber of a transwell assay (Neuro Probe Inc., Gaithersburg, MD, USA) and incubated for 15 h. To observe the cells that migrated into the lower chamber, the transwell membranes were fixed and stained using a Diff-Quik Staining Kit (Sysmex, Kobe, Japan) and cells on the undersurface of the membrane were counted under a light microscope. For the invasion assay, cells were plated in the upper compartments of the BioCoat Matrigel Invasion Chambers (BD Biosciences) for 24 h. The invading cells in the lower chamber were fixed, stained with the Diff-Quik Staining Kit and counted under a light microscope.

### Orthotopic tumour xenografts and histopathological analysis

All animal experiments were approved and performed in accordance with the Hanyang University Animal Care and Use Committee (Seoul, Korea). Five-week-old female NOD/SCID mice were purchased from the Korea Research Institute of Bioscience and Biotechnology. For measurement of *in vivo* tumour-initiating ability, breast cancer cells resuspended in Matrigel were injected into the mammary fat pads of the mice at limiting dilution. For xenografts with T47D cells, mice were supplemented with estradiol pellets (0.72 mg, released over 60 days; Innovative Research of America, Sarasota, FL, USA) 1 week before cell injection. The mice injected with Tet-inducible DOT1L shRNA-expressing MDA-MB-231 cells were administered 2 mg ml^−1^ DOX in 1% sucrose drinking water for induction of DOT1L knockdown. Tumour formation was monitored twice per week for 5–6 weeks. The proportion of tumour-initiating frequency was calculated using the L-Calc software (StemCell Technologies, Vancouver, BC, Canada, http://www.stemcell.com). The tumour size was calculated as follows: Volume (mm^3^)=(*a* × *b*^2^)/2, where *a* indicates the largest diameter and *b* is the perpendicular diameter. To investigate the functional properties of DOT1L in a tumorigenesis model, 1 × 10^6^ MCF10A cells expressing either DOT1L or an activated form of HRAS and co-expressing DOT1L and HRAS were injected into the mammary fat pads of the mice and tumour formation and growth were observed for 3 months as above. For the *in vivo* xenograft model of breast cancer metastasis, 1 × 10^5^ Tet-inducible DOT1L shRNA-expressing MDA-MB-231 cells and its control cells were orthotopically injected into the NOD/SCID mice. After 7 days of cell injection, the mice were administered 2 mg ml^−1^ DOX as described above and the tumour size was monitored. Three months after injection when tumours reached more 1,500 mm^3^, the mice were killed and lung metastatic colonization was monitored and quantified by histological analysis. For analysis of DOT1L and/or E-cadherin expression *in vivo*, the immunohistochemical staining was performed using consecutive sections of tumours from xenografted mice as described in the TMA and immunohistochemical staining section.

### Statistical analysis

Statistical significance of the differences between controls and experimental groups was calculated with the unpaired Student's *t*-test using the SPSS (version 12.0; SPSS Inc., Chicago, IL, USA) or Excel (Microsoft, Redmond, WA, USA) software packages. The *χ*^2^-test was used to examine the correlation of DOT1L with clinicopathological data variables in the human samples. The Kaplan–Meier method with log-rank or Gehan–Breslow–Wilcoxon test and Cox's proportional hazard regression model were used to calculate overall survival and disease-free survival. For multiple group comparison of *in vivo* data, repeated measures analysis of variance followed by least significant difference (LSD) *post hoc* test was performed. A *P* value<0.05 was considered to indicate statistical significance.

## Additional information

**How to cite this article:** Cho, M.-H. *et al.* DOT1L cooperates with the c-Myc-p300 complex to epigenetically derepress *CDH1* transcription factors in breast cancer progression. *Nat. Commun.* 6:7821 doi: 10.1038/ncomms8821 (2015).

## Supplementary Material

Supplementary Information Supplementary Figures 1-11 and Supplementary Tables 1-2

## Figures and Tables

**Table 1:** DOT1L accelerates *in vivo* tumour-initiating ability.

**Cell type**	**Cell number for injection**	**Days**			
		**7 Days**	**14 Days**	**21 Days**	**28 Days**
T47D CON	1,000	0/6	0/6	0/6	0/6
	5,000	0/7	0/7	0/7	1/7
	10,000	0/7	0/7	1/7	4/7
	50,000	0/7	3/7	4/7	5/7
					
TIC frequency			1/127,031 (1/41,695–1/387,021)	1/68,773 (1/28,585–1/165,461)	1/27,645 (1/13,800–1/55,381)
T47D DOT1L	1,000	0/6	0/6	1/6	1/6
	5,000	0/7	0/7	6/7	7/7
	10,000	0/7	4/7	7/7	7/7
	50,000	5/7	6/7	7/7	7/7
					
TIC frequency		1/63,976 (1/27,171–1/150,653)	1/24,116 (1/12,174–1/47,773)	1/2,752* (1/1,421–1/5,327)	1/1,977* (1/960–1/4,072)
*P* value				<0.0001	<0.0001

		**Days**			
		**14 Days**	**21 Days**	**28 Days**	**35 Days**
MDA-MB-231	500	0/6	0/6	1/6	3/6
shCON	1,000	0/6	0/6	3/6	5/6
	5,000	0/6	1/6	3/6	6/6
	10,000	1/6	3/6	5/6	6/6
TIC frequency		1/93,911 (1/13,382–1/659,030)	1/20,038 (1/7,582–1/52,961)	1/4,575 (1/2,383–1/8,780)	1/625 (1/300–1/1,301)
MDA-MB-231	500	0/6	0/6	0/6	1/6
shDOT1L	1,000	0/6	0/6	0/6	2/6
	5,000	0/6	0/6	1/6	3/6
	10,000	0/6	1/6	2/6	4/6
TIC frequency			1/93,911 (1/13,382–1/659,030)	1/28,615* (1/9,266–1/88,375)	1/6,427* (1/3,252–1/12,856)
*P* value				0.0058	0.0001

CON, control; DOT1L, Disruptor of telomeric silencing-1-like; NOD/SCID, non-obese diabetic/severe combined immunodeficient; TIC, tumour-initiating cell.

Limiting dilution assay. From 1,000 to 50,000 cells, T47D CON cells or T47D DOT1L cells and from 500 to 10,000 cells, MDA-MB-231 shCON cells or MDA-MB-231 shDOT1L cells were injected into the fat pad of NOD/SCID mice. The TIC frequency was calculated using the L-Calc software (Stemcell Tech, Vancouver, Canada, http://www.stemcell.com).

^*^P<0.05 versus CON (two-sided *t*-test).
