# Supplementary material for: DOT1L cooperates with the c-Myc-p300 complex to epigenetically derepress CDH1 transcription factors in breast cancer progression
Source: Nat Commun. 2015 Jul 22;6:7821. doi: 10.1038/ncomms8821 (PMC4525167; doi:10.1038/ncomms8821)
Supplement: Supplementary Information — Supplementary Figures 1-11 and Supplementary Tables 1-2 [file ncomms8821-s1.pdf]

Supplementary Figures

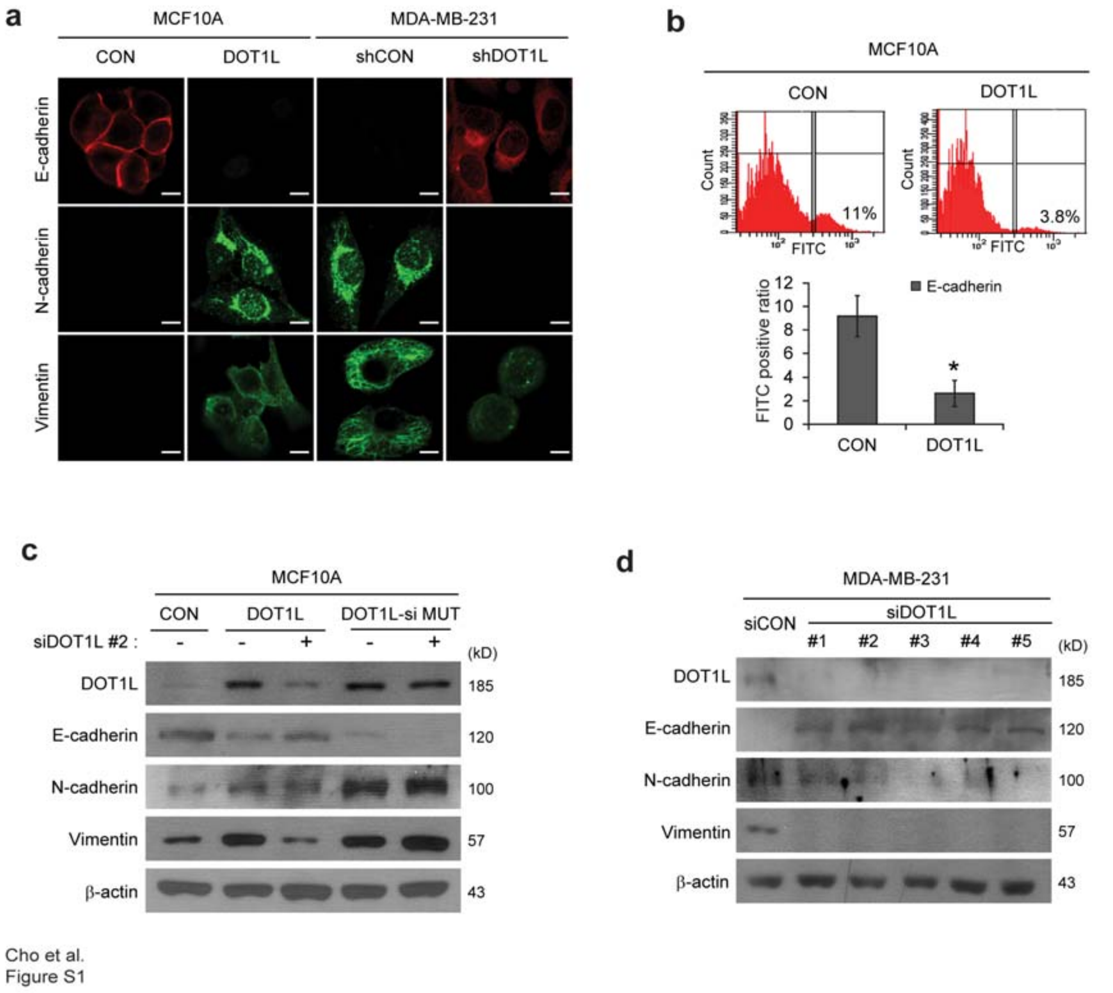

Supplementary Figure 1| DOT1L regulates the expression of epithelial and mesenchymal markers. (**a**) The expression levels and cellular localizations of EMT markers were confirmed by immunofluorescence staining. (**b**) Flow cytometric analysis of E-cadherin expression in DOT1L-overexpressing MCF10A cells. The histogram shows the percentage of the E-cadherin-expressing (Alexa Fluor 488-positive) cell population. Error bars represent means ± s.d. of triplicate measurements. *$P < 0.05$ *vs*. controls (Student's *t*-test). (**c**) Effect of DOT1L siRNA on epithelial and mesenchymal markers in MCF10A cells expressing DOT1L wild type or siRNA-resistant DOT1L mutant (si MUT) were examined by immunoblotting. (**d**) MDA-MB-231 cells were transiently transfected with DOT1L siRNAs and subjected to immunoblotting.

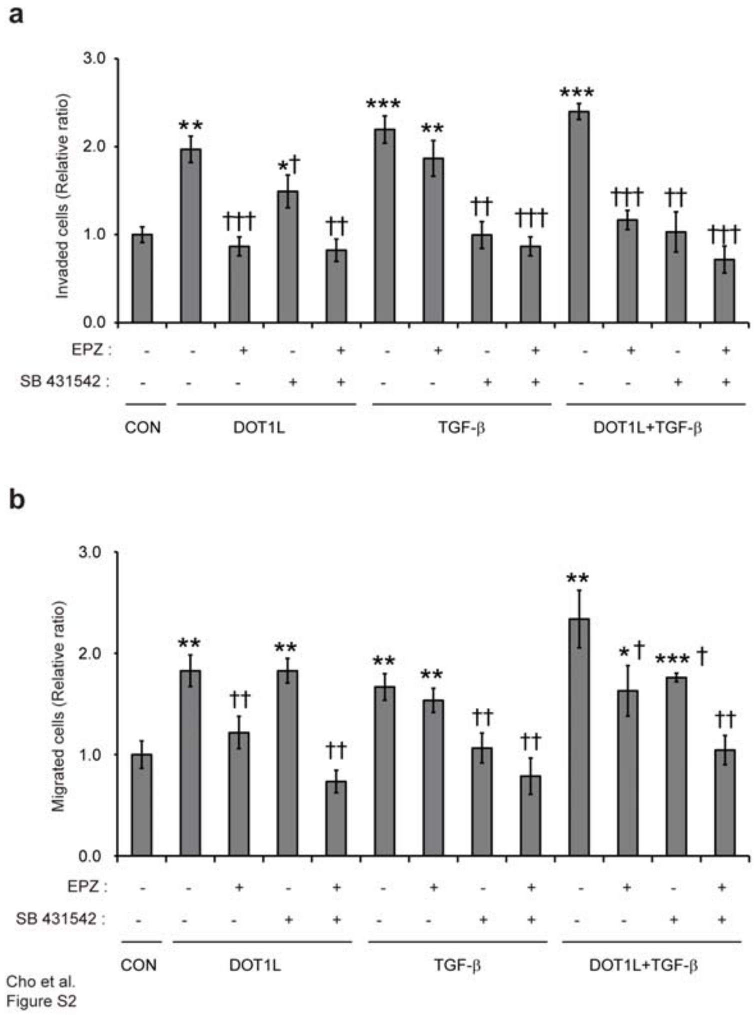

Supplementary Figure 2| Effect of TGF-β on DOT1L-induced breast cancer migration and invasion. (**a**,**b**) DOT1L-overexpressing MCF10A cells and its control cells in the presence or absence of 100 pM TGF-β1 were treated with 10 μM TGF-β inhibitor SB 431542, 1 μM DOT1L inhibitor EPZ004777 (EPZ), or both for 48 h, and invasion (a) and migration (b) by the indicated cells were analyzed and quantified. Results are shown as means ± s.d. of experiments in triplicate. *$P < 0.05$, **$P < 0.01$, *$P < 0.001$, *vs*. CON (lane 1); †$P < 0.05$, ††$P < 0.01$, †††$P < 0.001$, *vs*. vehicles (lanes 2, 6, and 10) by Student's *t*-test.

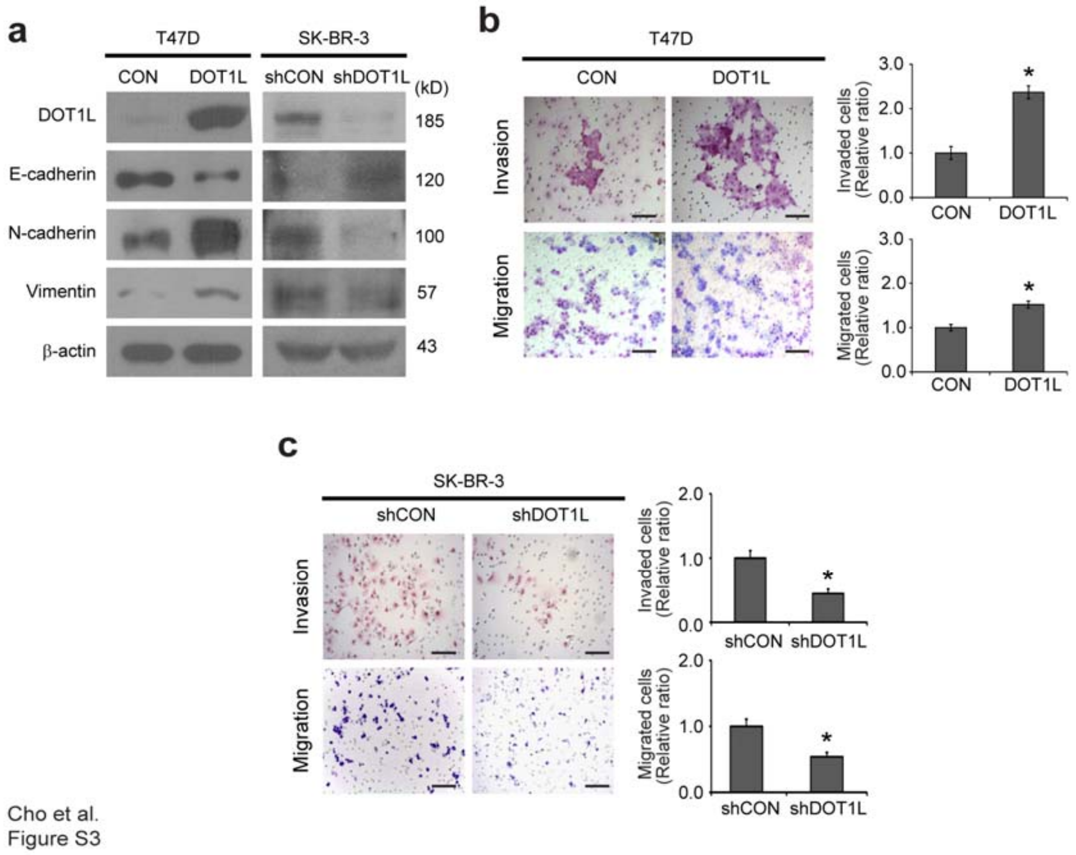

Supplementary Figure 3| Effect of DOT1L on EMT and invasion ability in human breast cancer cell lines. (**a**) The expression levels of epithelial and mesenchymal markers in DOT1L-overexpressing T47D or Tet-inducible DOT1L knockdown (shDOT1L) SK-BR-3 cell lines were analyzed using immunoblotting. (**b,c**) Representative images and quantification of invasion (upper) and migration (lower) by the indicated cells (b, T47D; c, SK-BR-3). The data represents the means ± s.d. of triplicate assays. *$P < 0.05$ compared with CON or shCON (Student's *t*-test).

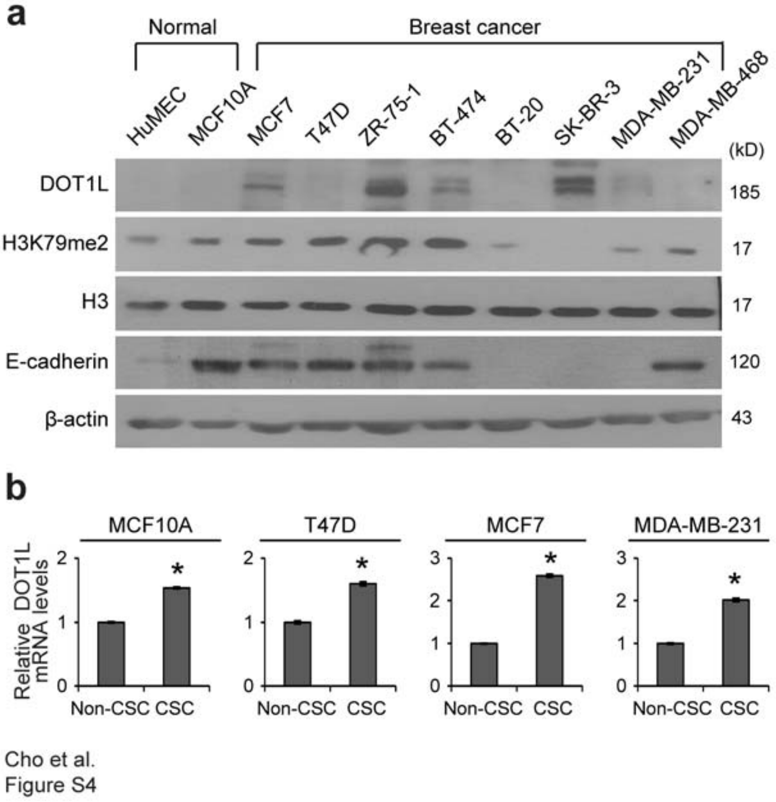

Supplementary Figure 4| DOT1L expression in breast normal and cancer cell lines and breast CSCs. (**a**) The expression levels of DOT1L, E-cadherin and H3K79me2 in HuMEC normal human epithelial cells, MCF10A immortalized non-cancer cells and eight breast cancer cell lines were analyzed by immunoblotting. (**b**) The breast CSC ($CD44^{+}/CD24^{-}/ESA^{+}$) and non-CSC (Non-CSC) subpopulations among MCF10A, T47D, MCF7 and MDA-MB-231 cells were sorted using FACS Aria and the DOT1L mRNA level in the indicated fractions was determined using qRT-PCR. The *18S* gene was used as an internal control for qRT-PCR. The data represents the means ± s.d. of triplicate assays. *$P < 0.05$ compared with Non-CSC (Student's *t*-test).

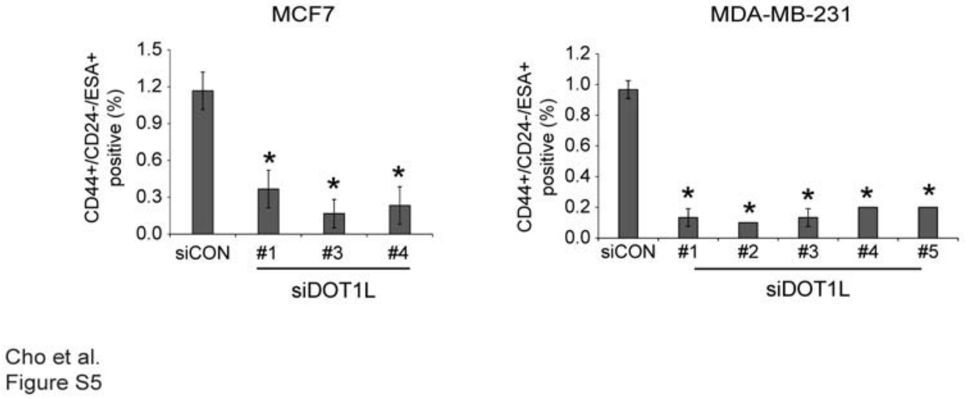

Supplementary Figure 5| Effect of DOT1L knockdown on breast CSC population. Cells were transfected with indicated DOT1L siRNAs for 48 h and subjected to FACS analysis for measurement of changes in the $CD44^+/CD24^-/ESA^+$ cell population. The data represents the means ± s.d. of triplicate assays. *P < 0.05 compared with siCON (Student's *t*-test).

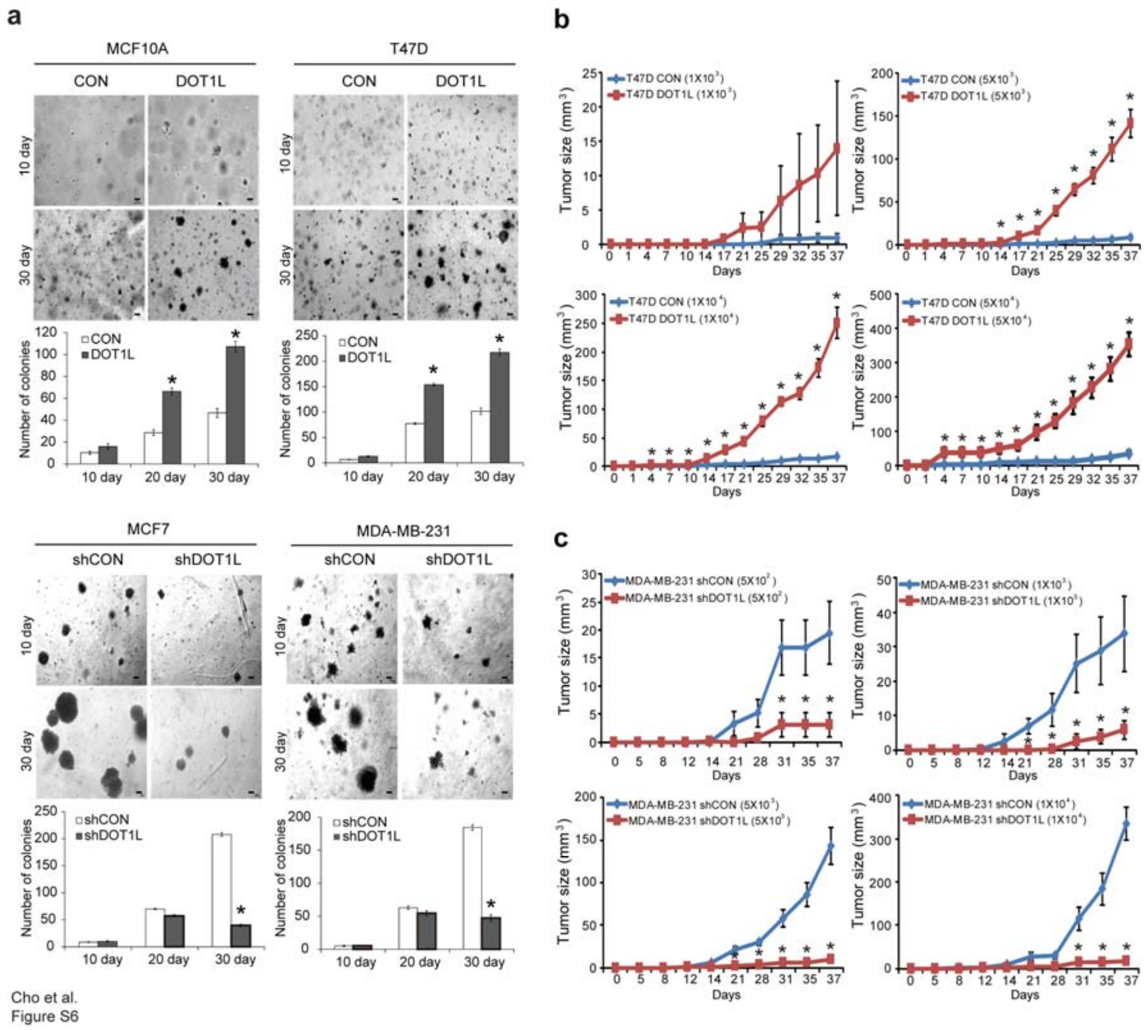

Supplementary Figure 6| DOT1L accelerates in vitro and in vivo breast tumor growth. (**a**) Soft-agar colony formation assay was performed to examine anchorage-independent tumor growth in stable DOT1L-overexpressing and Tet-inducible DOT1L shRNA-expressing cells. Results are shown as means ± s.d. of experiments in triplicate. Scale bars = 100 μm. **P* < 0.05 *vs*. controls (Student's *t*-test). (**b**) To assess the effect of DOT1L overexpression on *in vivo* tumor growth, NOD/SCID mice were orthotopically injected with control or DOT1L-overexpressing T47D cells, and the tumor size in each group was measured twice per week for 40 days. (**c**) The tumor growth rate in xenograft mice with Tet-inducible DOT1L-knockdown MDA-MB-231 cells was analyzed as described above. Error bars in b-c indicate the means ± s.e.m (n = 5). * $P < 0.05$ *vs*. controls (Student's *t*-test).

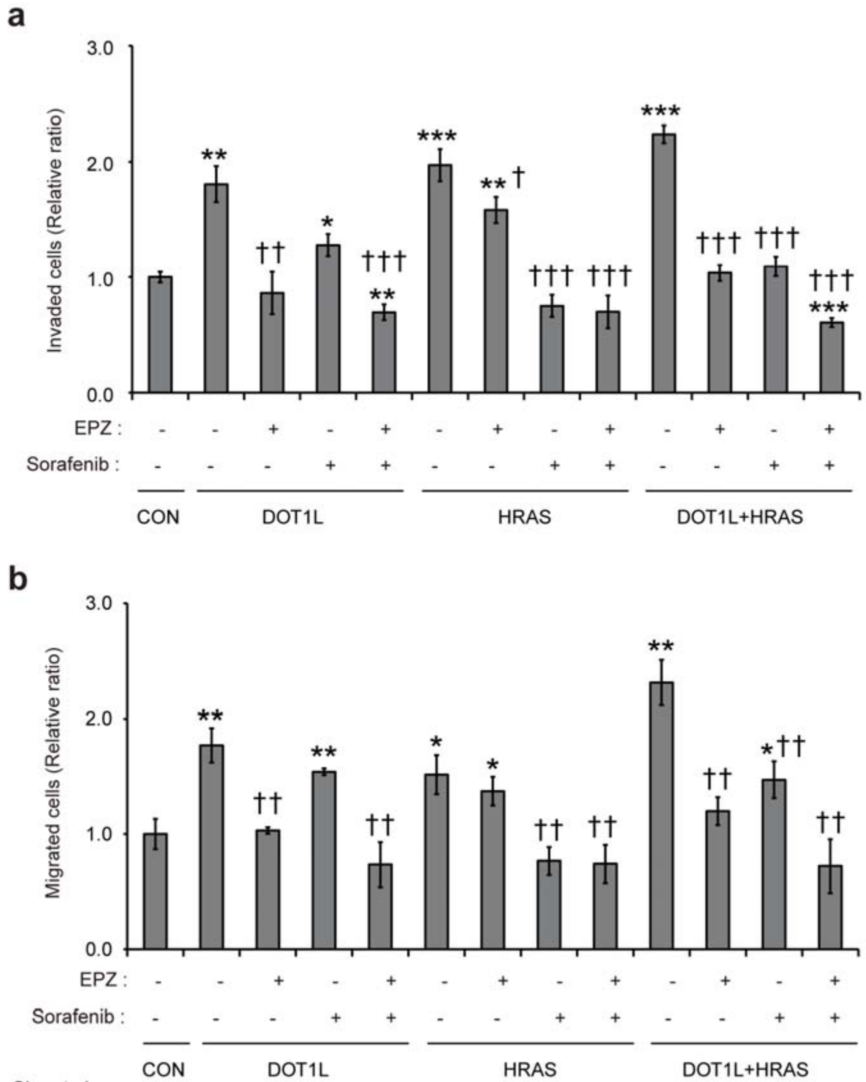

Cho et al.
Figure S7

Supplementary Figure 7| DOT1L and HRAS effect on breast cancer migration and invasion in MCF10A cells. (**a**,**b**) MCF10A cells expressing either DOT1L or HRAS and MCF10A cells coexpressing DOT1L and HRAS (DOT1L + HRAS) were treated with either 1 μM EPZ004777 (EPZ) for 48 h, or 20 μM RAF inhibitor Sorafenib for 24 h, or both inhibitors. The invasion (a) and migration (b) by the indicated cells were analyzed and quantified. Results are shown as means ± s.d. of experiments in triplicate. *$P < 0.05$, **$P < 0.01$, *$P < 0.001$, *vs*. CON (lane 1); †$P < 0.05$, ††$P < 0.01$, †††$P < 0.001$, *vs*. vehicles (lanes 2, 6, and 10) by Student's *t*-test.

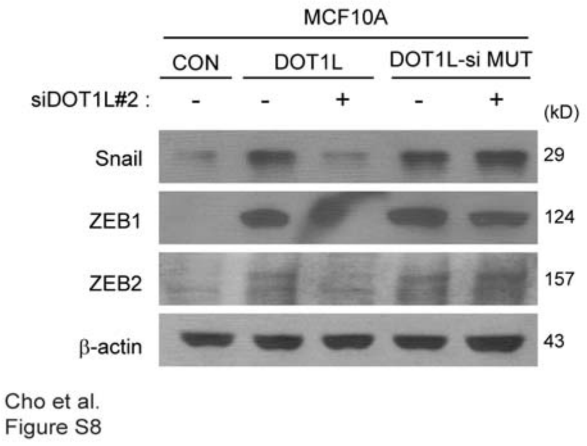

Supplementary Figure 8| DOT1L regulates EMT-TFs expression. (**a,b**) MCF10A cells expressing DOT1L wild type or siRNA-resistant DOT1L mutant (si MUT) were treated with DOT1L siRNA (#2) for 48 h. The lysates from indicated cells were then subjected to immunoblotting for analysis of EMT-TFs expression.

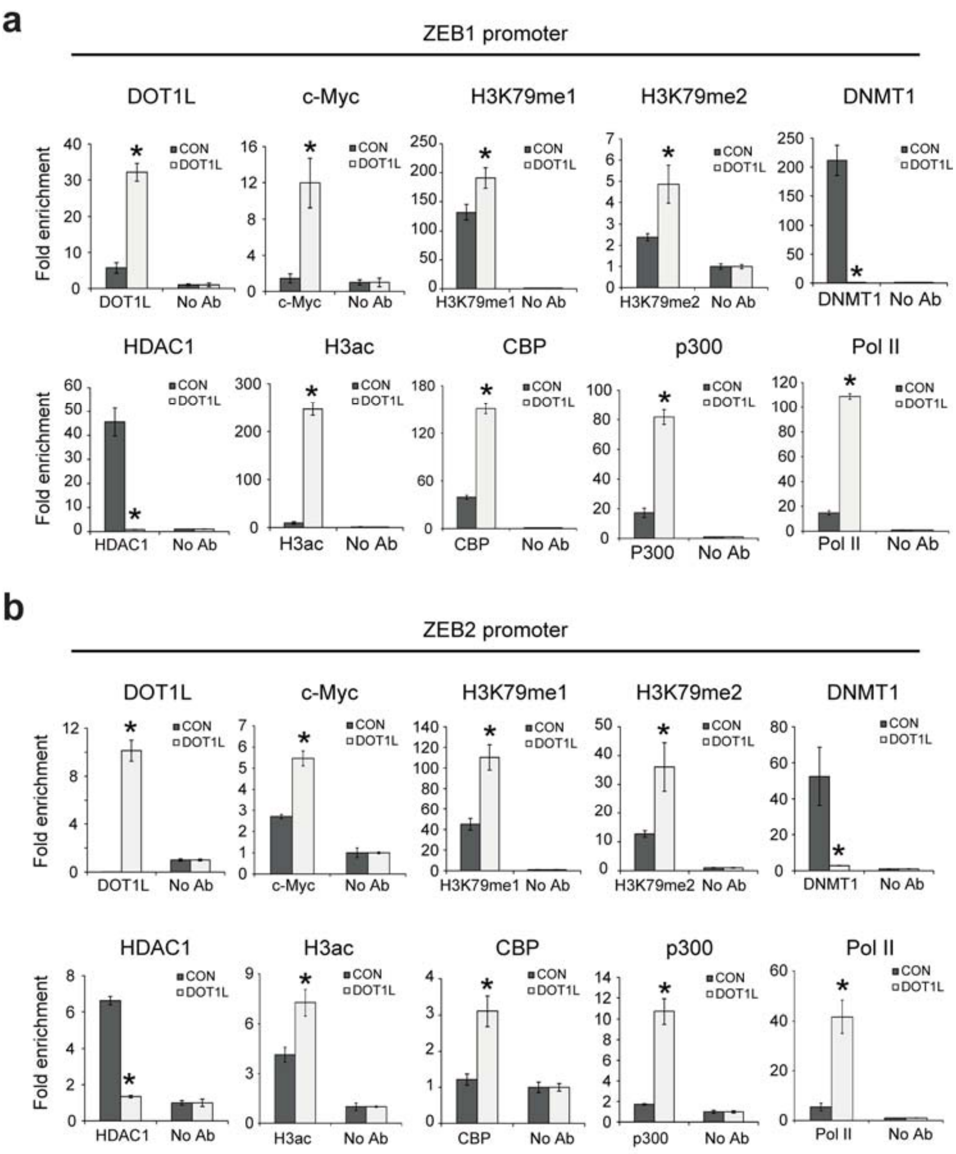

Cho et al.
Figure S9

Supplementary Figure 9| DOT1L epigenetically regulates ZEB1 and ZEB2 gene expression. (**a,b**) ChIP analysis showing the recruitment of indicated proteins and enrichment of histone marks in the promoter regions of *ZEB1* and *ZEB2* genes. Results are shown as means ± s.d. of experiments in triplicate. *$P < 0.05$ *vs*. CON (Student's *t*-test).

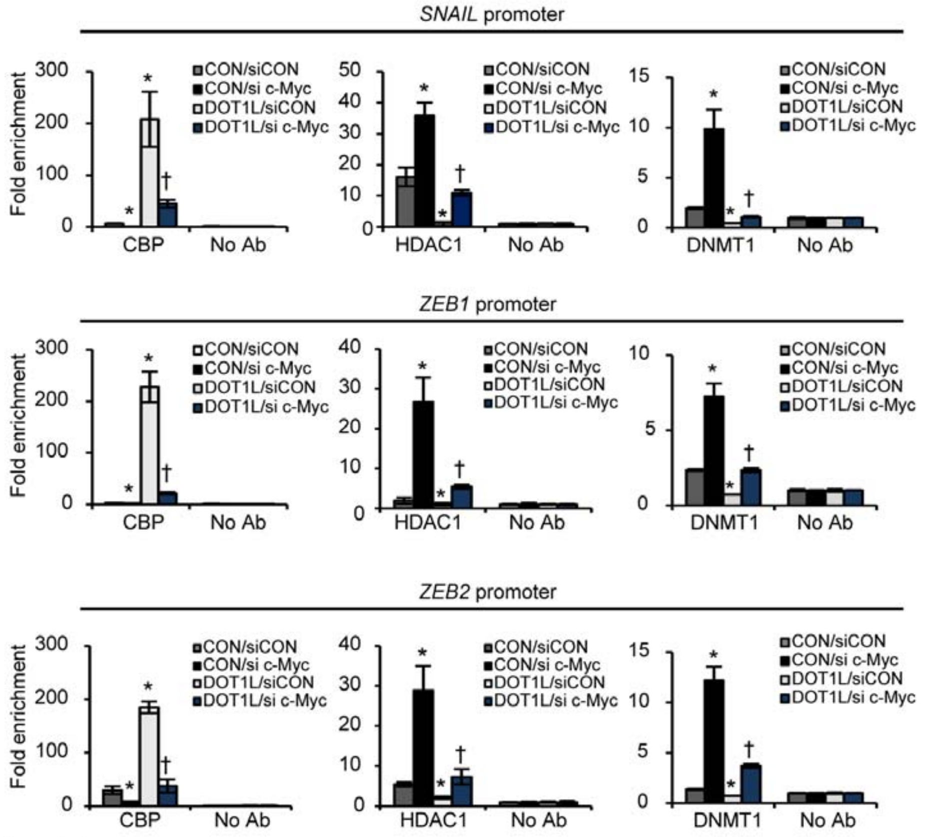

Cho et al.
Figure S10

Supplementary Figure 10| Effect of c-Myc on DOT1L-mediated epigenetic regulation of EMT-TFs. DOT1L-overexpressing or control MCF10A cells were transfected with c-Myc siRNA for 48 h. The dependency of EMT-TF regulation by DOT1L on c-Myc was then analyzed by ChIP-qPCR assay in these cells. Results are shown as means ± s.d. of experiments in triplicate. * and †, $P < 0.05$ *vs*. CON/siCON and DOT1L/siCON, respectively (Student's *t*-test).

Full unedited blots for Figure 1

Fig. 1d

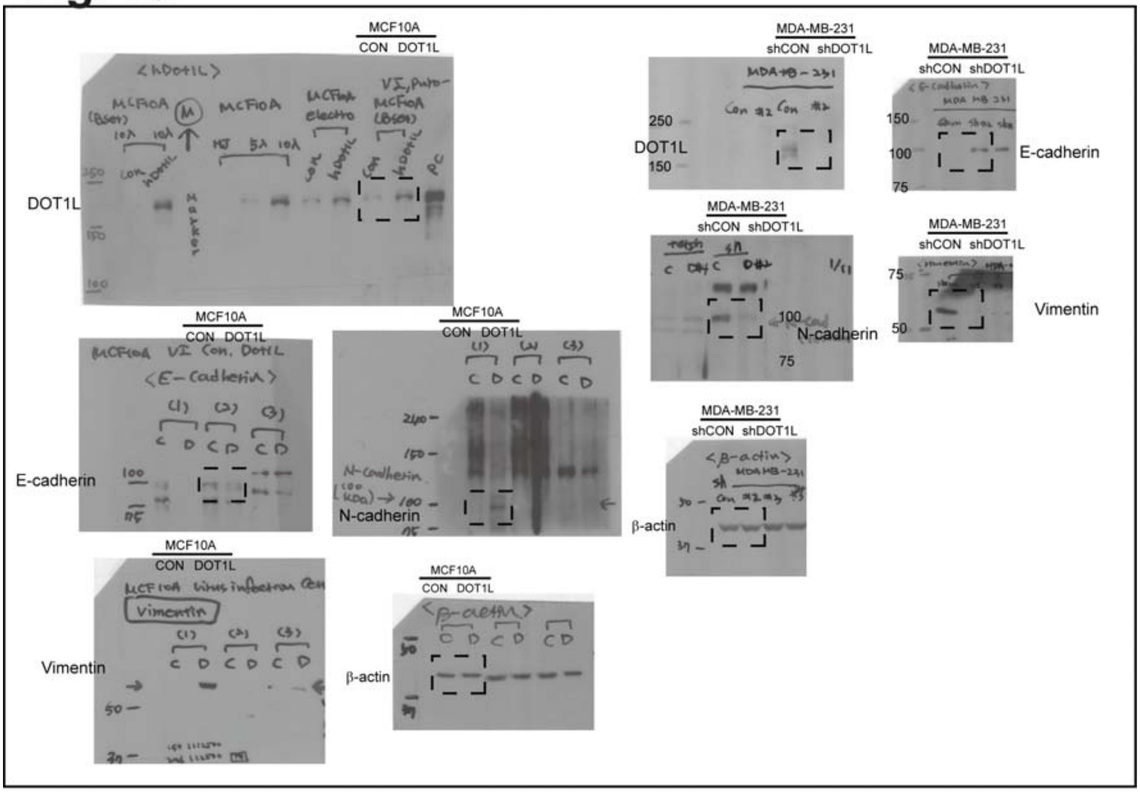

Fig. 1e

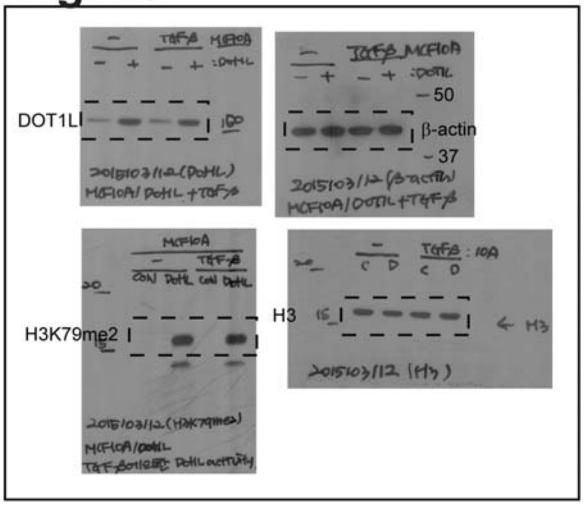

Supplementary Figure 11| Original images of Western blots –for Figure 1

Full unedited blots for Figure 2

Fig. 2a

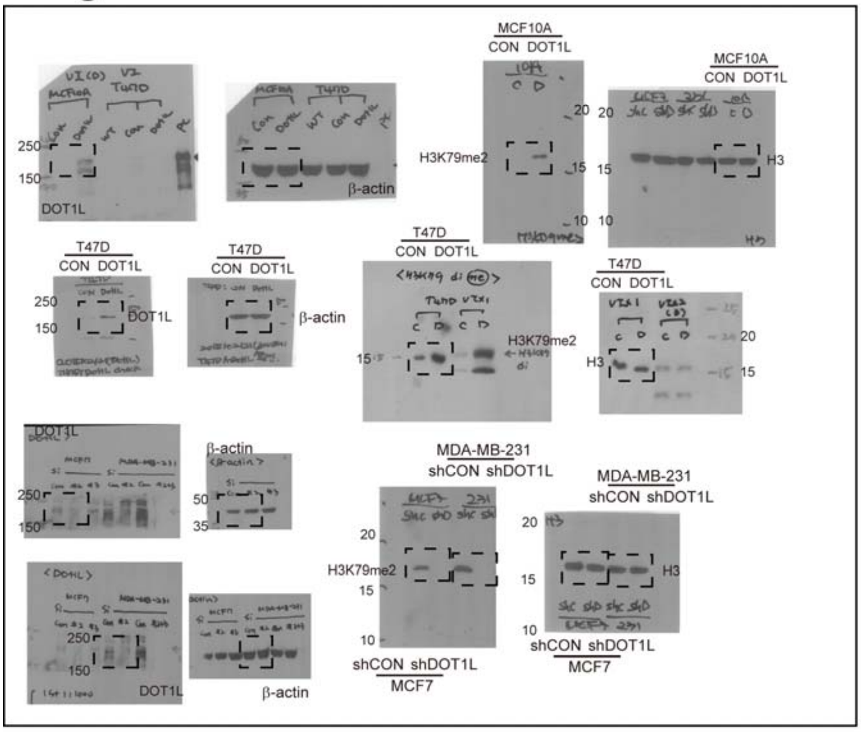

Fig. 2c

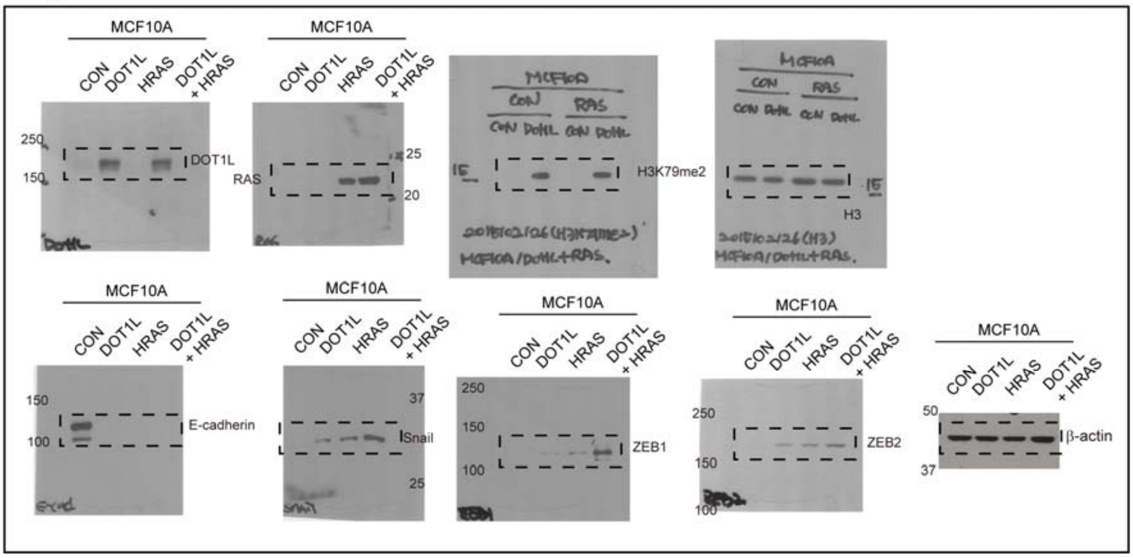

Supplementary Figure 11 (cont.)| Original images of Western blots –for Figure 2

Full unedited blots for Figure 3

Fig. 3c

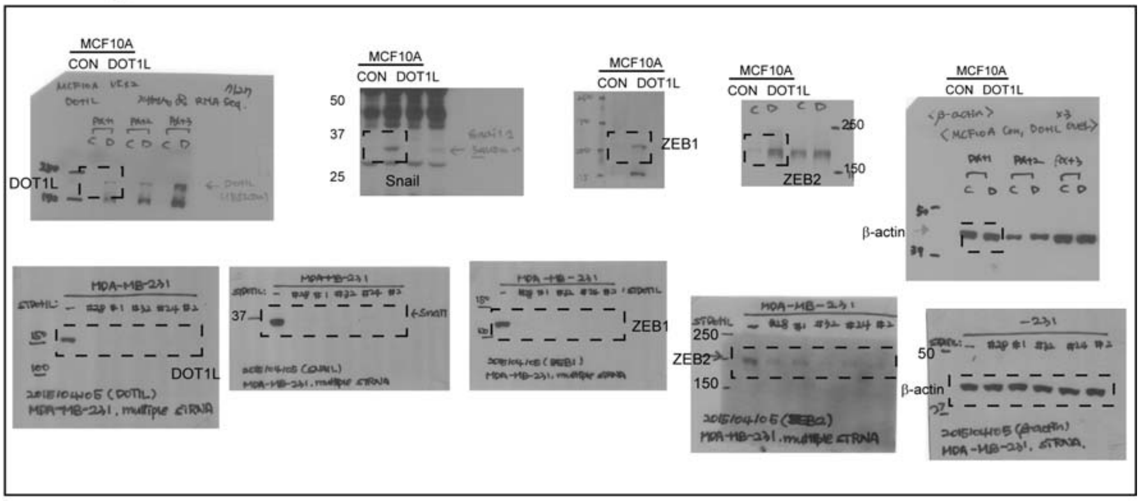

Fig. 3g

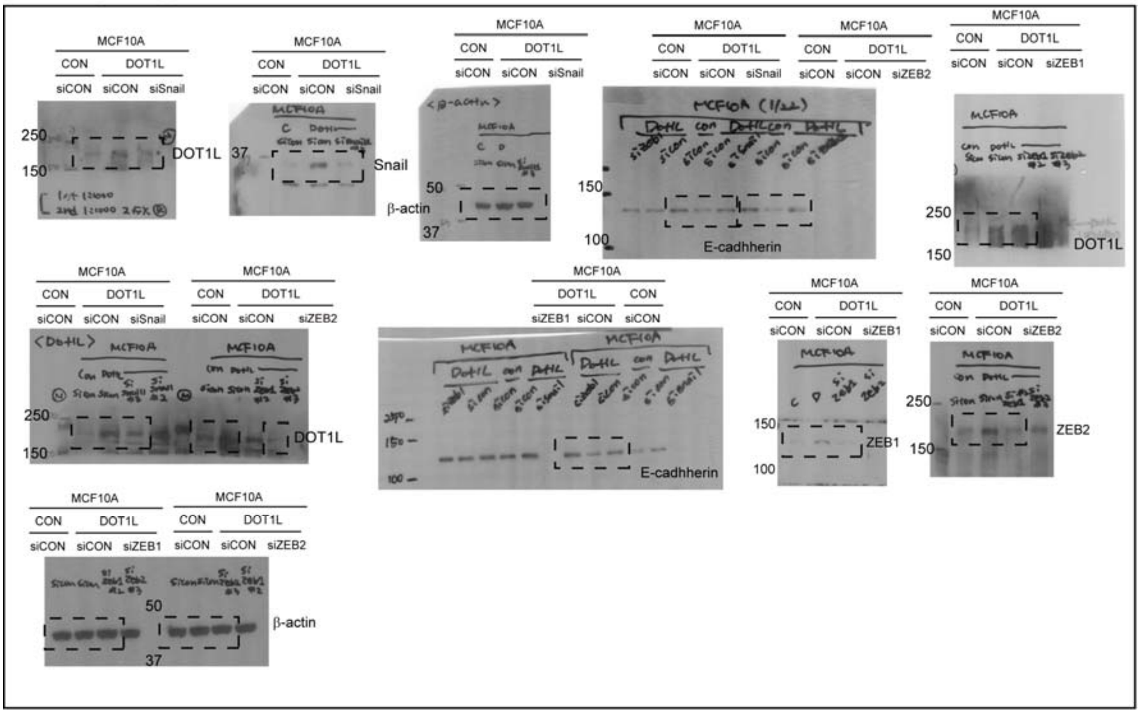

Supplementary Figure 11 (cont.)| Original images of Western blots –for Figure 3

Full unedited blots for Figure 4

Fig. 4a

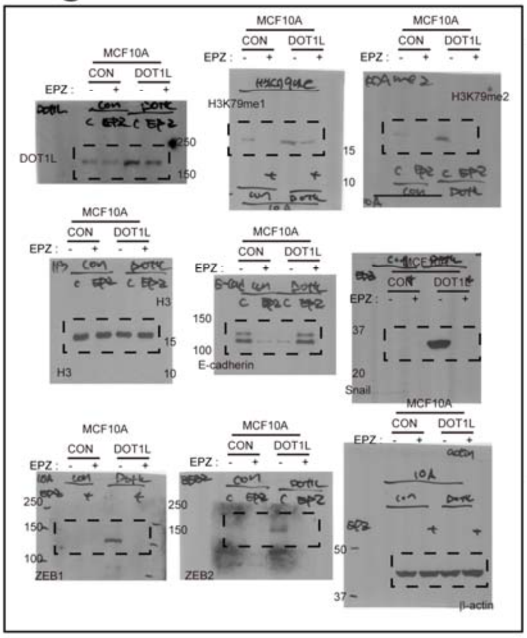

Fig. 4c

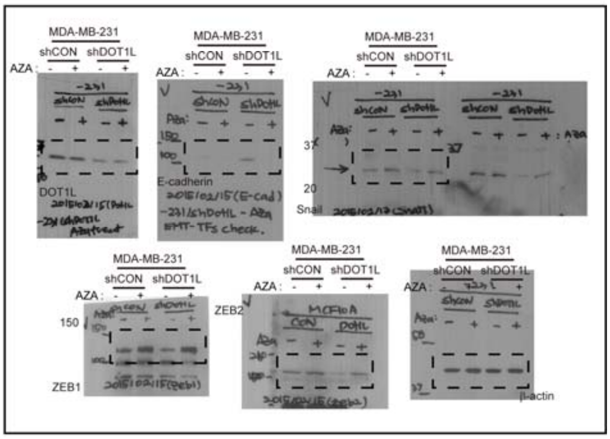

Fig. 4d

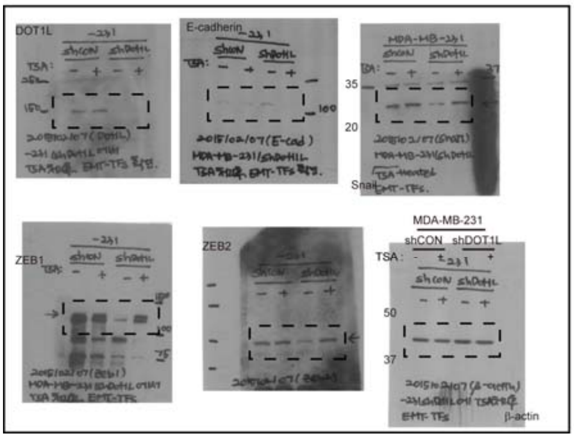

Fig. 4e

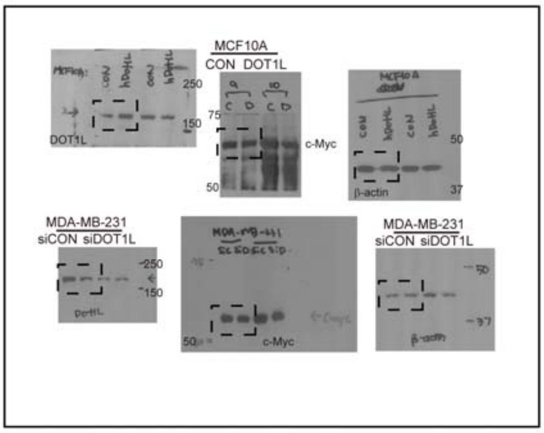

Supplementary Figure 11 (cont.)| Original images of Western blots –for Figure 4a-e

Full unedited blots for Figure 4

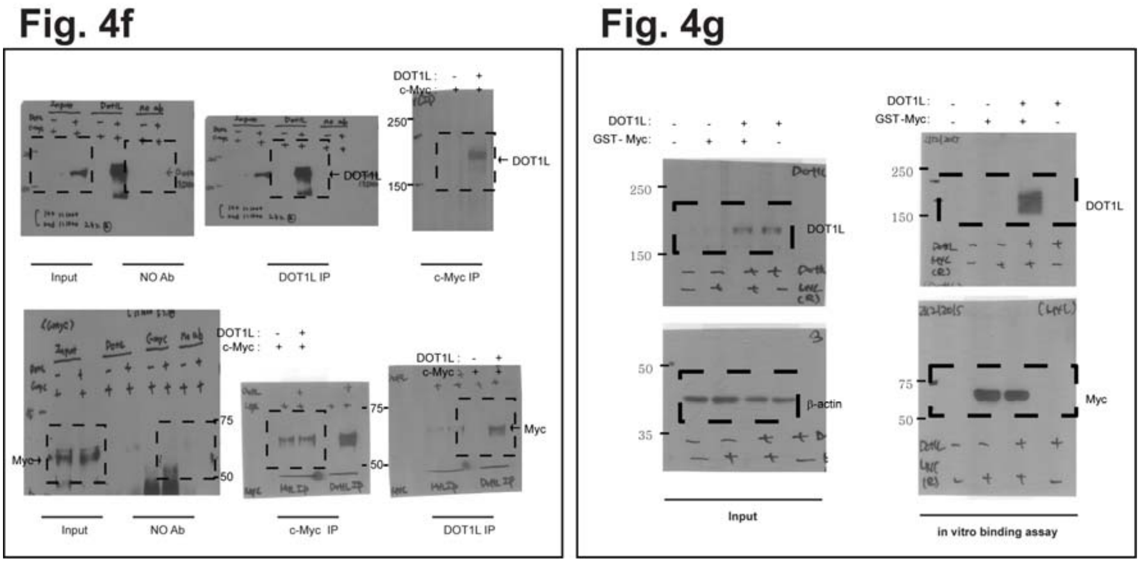

Supplementary Figure 11 (cont.)| Original images of Western blots –for Figure 4f,g

Full unedited blots for Figure 5

Fig. 5a

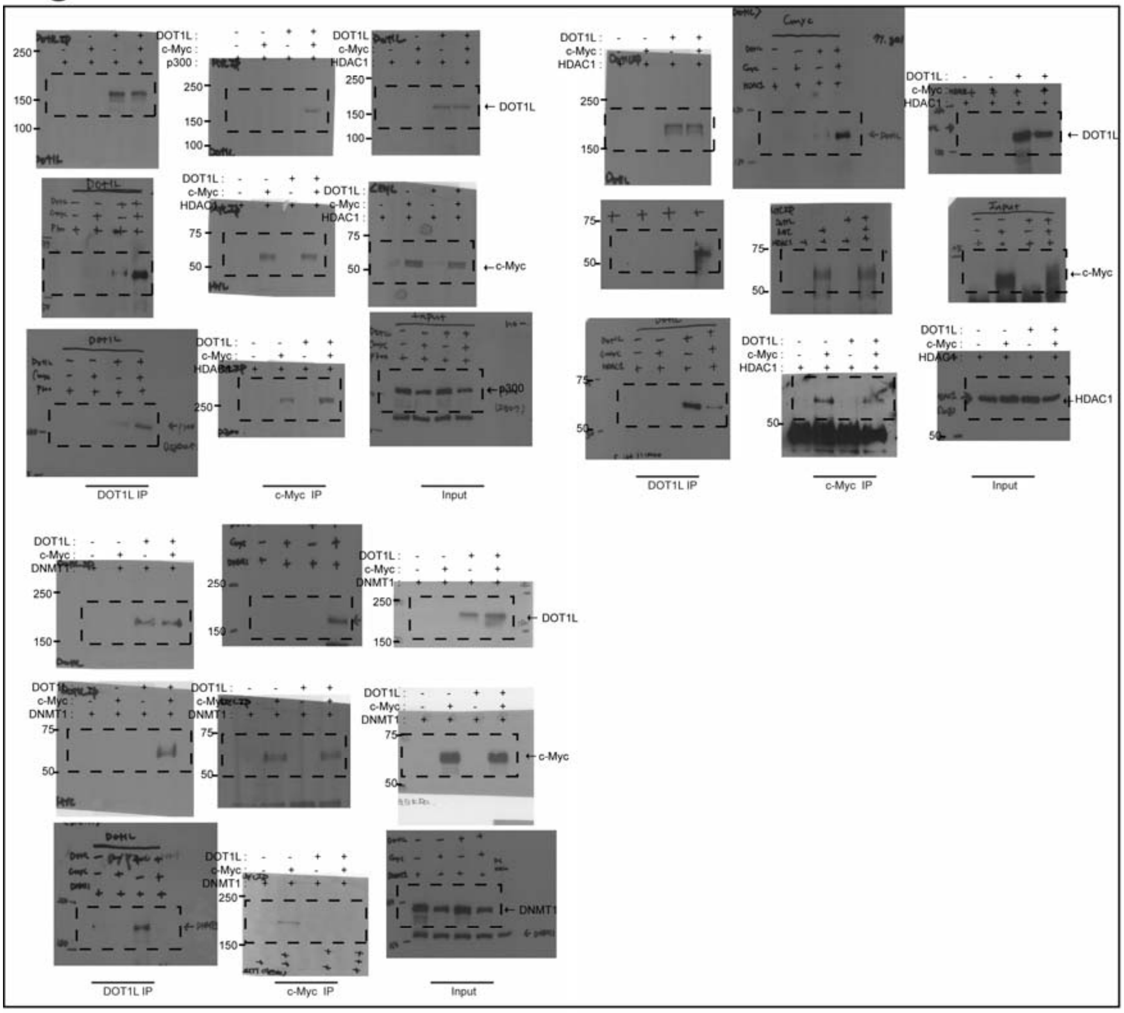

Fig. 5b

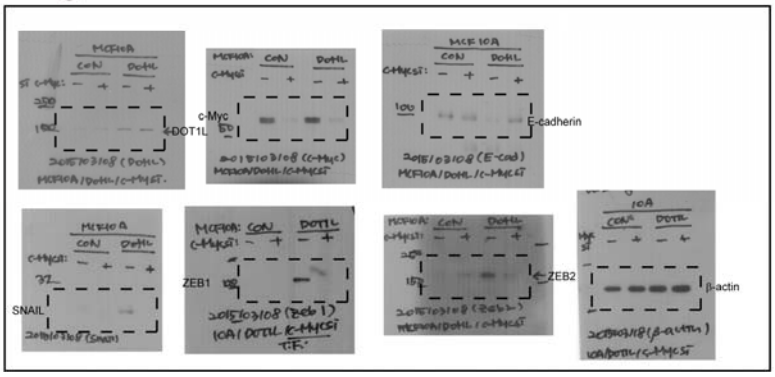

Supplementary Figure 11 (cont.)| Original images of Western blots –for Figure 5

Supplementary Table 1. Correlation of DOT1L expression with clinicopathologic data in breast cancers (*n* = 182)

Clinico-pathologic Parameters	*n*	DOT1L Expression		*P* value (χ^2-test)
		Negative	Positive	
AJCC Stage				0.223
Stage I-II	126	84 (66.7%)	42 (44.8%)	
Stage III-IV	55	30 (54.5%)	25(45.5%)	
Lymph node metastasis				**0.003**
Negative	86	64 (74.4%)	22 (25.6%)	
Positive	96	51 (53.1%)	45 (46.9%)	
Lymphatic invasion				**< 0.001**
Negative	78	61 (78.2%)	17 (21.8%)	
Positive	104	54 (51.9)	50 (48.1)	
Estrogen receptor				**0.046**
Negative	91	51 (56.0%)	40 (44.0%)	
Positive	91	64 (70.3%)	27(29.7%)	
Progesterone receptor				**0.014**
Negative	87	47(54.0%)	40 (46.0%)	
Positive	95	68 (71.6%)	27 (28.4%)	
HER2 (IHC)				0.897
Negative	53	33 (62.3%)	20 (37.7%)	
Positive	128	81 (63.3%)	47 (36.7%)	
Triple-negativity				**0.027**
Triple-negative	135	79 (58.5%)	56 (41.5%)	
Non-triple-negative	47	36 (76.6%)	11 (23.4%)	

Supplementary Table 2. Cox regression analysis of overall and disease-free survival of breast cancer patients according to DOT1L expression

	Total	ER+ *	ER- **
Overall survival			
P value	0.548	0.433	0.042
Hazard ratio	1.282	0.409	2.624
Disease free survival			
P value	0.968	0.467	0.184
Hazard ratio	1.013	0.760	1.446
Number of patients	182	91	91

*ER+ and **ER-, estrogen receptor-positive and -negative breast cancer patients, respectively
